# Prevalence, reasons, and determinants of dietary supplements use among undergraduate female students of health and non-health colleges in a Saudi public university

**DOI:** 10.1371/journal.pone.0247295

**Published:** 2021-03-03

**Authors:** Md. Ashraful Islam, Aseel Fuad Al-karasneh, Mehwish Rizvi, Zeb-Un Nisa, Ahmed Majed Albakheet, Mohammed Abdullah Alshagawi, Muhammad Shahid Iqbal, Abdullah Isa Almuzel, Hani Sadiq Al Afif, Mansour Adam Mahmoud, Alnada Abdalla Mohamed Ibrahim, Mohammad Akbar Hossain, Muhammad Bilal Maqsood, Atta Abbas Naqvi, Abdul Haseeb, Shazia Jamshed

**Affiliations:** 1 Department of Pharmacy Practice, College of Clinical Pharmacy, Imam Abdulrahman Bin Faisal University, Dammam, Saudi Arabia; 2 Dow College of Pharmacy, Dow University of Health Sciences, Karachi, Pakistan; 3 Faculty of Pharmacy, Ziauddin University, Karachi, Pakistan; 4 Department of Pharmacology, College of Clinical Pharmacy, Imam Abdulrahman Bin Faisal University, Dammam, Saudi Arabia; 5 Department of Clinical Pharmacy, College of Pharmacy, Prince Sattam bin Abdulaziz University, Alkharj, Saudi Arabia; 6 College of Clinical Pharmacy, Imam Abdulrahman Bin Faisal University, Dammam, Saudi Arabia; 7 Department of Clinical and Hospital Pharmacy, College of Pharmacy, Taibah University, Al-Madinah Al-Munawarah, Saudi Arabia; 8 Department of Pharmacy practice, College of Pharmacy, Princess Nourah bint Abdulrahman University, Riyadh, Saudi Arabia; 9 Department of Pharmaceutics, Faculty of Pharmacy, University of Khartoum, Khartoum, Sudan; 10 Department of Pharmacology and Toxicology, College of Medicine, Umm Al Qura University, Makkah, Saudi Arabia; 11 King Abdullah International Medical Research Center, Al Ahsa, Saudia Arabia; 12 Swiss Business School, Kloten, Zurich, Switzerland; 13 Department of Pharmacy, Ministry of National Guard Health Affairs, AlAhsa, Saudi Arabia; 14 King Saud bin AbdulAziz University for Health Sciences, Al Ahsa, Saudi Arabia; 15 Department of Clinical Pharmacy, College of Pharmacy, Umm Al Qura University, Makkah, Saudi Arabia; 16 Department of Pharmacy Practice, Faculty of Pharmacy, UniSZA, Kuala Terengganu, Malaysia; 17 Qualitative Research-Methodological Application in Health Sciences Research Group, Kulliyyah of Pharmacy, International Islamic University Malaysia, Kuantan, Malaysia; Jouf University, Kingdom of Saudi Arabia, SAUDI ARABIA

## Abstract

**Purpose:**

The aim of this study was to gather data from female students studying in both health and non-health colleges at Imam Abdulrahman Bin Faisal University and report the prevalence, reasons, and determinants of dietary supplements use.

**Methods:**

A month-long cross-sectional study was conducted in health and non-health colleges affiliated to Imam Abdulrahman Bin Faisal University in Dammam, Saudi Arabia. Convenient sampling was employed, and the data was gathered through an online survey using the English and Arabic versions of the Dietary Supplement Questionnaire (DSQ). The data was analyzed using SPSS version 23 and Medcalc. The study was approved by an ethics committee.

**Results:**

Data from 545 participants was collected. The overall prevalence of dietary supplement use was 32.7% (95% CI: 29.06%– 36.51%). The prevalence was 29.77% (95% CI: 25.29%– 34.56%) among students at all health colleges combined and, it was 37.50% (95% CI: 31.36%– 43.96%) among students at all non-health colleges. Most students used a brand product, spent a monthly cost of SAR 286 (USD 76.3) on supplements and agreed that supplements were good for health (N = 392, 71.9%). Students from non-health- colleges agreed that dietary supplements are good for health in greater numbers as compared to non-health college students (p < 0.001). Students aged ≥ 20 years, studying in a non-health college and up to 3^rd^ year of study, were more 2 times more likely to agree that dietary supplements are good for health.

**Conclusion:**

Supplements were commonly used among female students at this university however, it was quite low as compared to students from other local and regional universities. Prevalence was higher in non-health colleges as compared to health colleges and the most commonly used supplements were brand products and, multivitamins, used for general health and well-being. This highlights the inclination of students towards supplement use.

## Background

Adequate nutrition is essential for maintaining health and well-being. Apart from nutrition intake through diet, supplements are commonly used to replenish the body with essential nutrients that are important in regulating the body’s metabolic processes [1]. Dietary supplements (DS) are available as pharmaceutical dosage forms that contain essential vitamins, minerals, oils, extracts and natural products. It may also contain a combination of these nutrients [2, 3]. These pharmaceutical dosage forms may be a brand which is a research-based pharmaceutical product patented for a certain period of time, or generic form that are prepared after the patent of a brand product has expired. Since generic forms are not research-based they are less expensive than brands [3]. The use of DS may be for general nutritional purposes or for specific purpose such as sports, high endurance exercise, etc. They may be used in pregnancy, aging and, prevention of diseases. The benefits of DS are evident when used in recommended dose and as directed [2, 3].

Evidence highlights that dietary supplements are commonly used in the developed countries such as the US and the UK [3, 4]. For instance, the United States is the biggest market for DS and data from the third National Health and Nutrition Examination Survey (1988–1994) (NHANES III) reported that 40% of population in the US used supplements while NHANES 1999–2000 data reported the figure of 52% for the same [5, 6]. According to the Transparency Market Research, the value of dietary supplement market in US is expected to amount to USD 278.96 billion in 2021 [7]. Several studies highlighted the perceptions of the general public that supplements are beneficial and may prevent chronic illnesses [4, 8, 9]. However, there are negative effects with their use such as adverse drug events (ADEs), adverse drug reactions (ADRs) and interaction of supplements with either drug or food that may pose health hazard [10]. Patients with some forms of illnesses such as those with kidney damage requiring dialysis and taking vitamin D and calcium over a large period of time may develop calcification in their soft tissues [11–14].

Epidemiological studies mention that demographic characteristics may act as determinants of supplement use [5, 6, 15]. Few notable demographic traits associated with increased DS use are old age, female gender, level of education, etc. [5]. It was observed in a study that college students were more frequent users of supplements as compared to the general population [16]. In addition, studying a health and/or non-health subject may have some sort of effect on DS use [3, 17].

Apart from the North American market, Asia Pacific region is ranked as the second biggest market for DS [5]. Saudi Arabia is located in the Middle East and is the biggest market for DS in this region [3]. The estimated worth of DS in Saudi Arabia is over USD 2 billion and DS accounts for over 4% of all pharmaceuticals sold in the country [3, 7].

Evidence from a Saudi university highlights that DS use was more common among male students who studied in health colleges [3]. However, previous studies reported prevalence of DS only and further research to delve into the reasons and determinants of DS use are needed [18, 19] Data from the female students at a public sector university in Riyadh region highlighted an increased use of supplements [19]. Another study at this study venue reported a detailed account of prevalence of supplements use in colleges, years of study etc., along with students’ attitudes towards the DS use and the monthly cost attributed to their use [3]. However, the findings were limited to male students only. Therefore, a need was felt to report the same from female students enrolled at this venue. This study followed the methodology of Naqvi and colleagues [3]. It gathered data from female students studying in colleges of both health and non-health subjects affiliated to this university. Hence, the aim was to report the prevalence, reasons, and determinants of dietary supplements use among undergraduate female students of health and non-health colleges at Imam Abdulrahman Bin Faisal University (IAU).

## Methods

This study was designed as a cross-sectional survey and was conducted for a month (July 2020) at IAU.

### Venue of study

All female campuses of the ten health and non-health colleges at Imam Abdulrahman Bin Faisal University in Dammam, Saudi Arabia, served as venues. The health colleges included colleges of pharmacy, medicine, nursing, applied medical science and dentistry. The non-health colleges included colleges of design, engineering, applied studies and community services, business, and science.

### Participants and eligibility criteria

All female undergraduate students who were currently enrolled and studying in any of the above mentioned colleges of the university and, who were willing to participate in the study, were identified as target participants. Students who had either graduated, or dropped their studies and, those who did not consent to participate, were excluded.

### Sampling strategy and data collection

A convenient sampling strategy was followed, and students were approached via an online survey. The survey link was distributed through most commonly used social media platforms such as talk and text messaging applications, emails, short message service, etc. A focal person was chosen from each college on a voluntary basis and was appraised regarding the study objectives. The focal person was assigned the task of briefing the students and collecting data. Students who consented to participate were asked to sign an electronic informed consent. After signing of the consent form, they could begin filling their responses in the electronic questionnaire.

### Sample size calculation

The sample size was calculated based on the total number of currently enrolled students at the university. Based on the official university figures, the number of active students was 14332 [20]. Hence, this figure was the target population. The sample size was calculated using an online calculator [21]. Considering an error rate of 5% and a confidence level of 95%, the required sample size was 374. However, we aimed to gather as many responses as possible from these colleges.

### Research instrument

The Arabic and English versions of the Dietary supplement questionnaire (DSQ) were used to gather students’ responses [2, 3]. The DSQ was previously developed and validated by Naqvi and colleagues in undergraduate students. It was first formulated in English and Urdu languages and was validated in Pakistani undergraduate students. Later, it was translated into Arabic language and was validated in Saudi undergraduate male students [2, 3]. The questionnaire was piloted in 18 students before actual data collection. The pilot data was not included in the final analysis.

### Data analyses and presentation

The data were analyzed using IBM SPSS version 23 (Armonk, NY). The data were expressed in frequencies (N) and percentages (%). The continuous data were expressed by mean (X) and standard deviation (SD). The prevalence was calculated using Medcalc [22]. The number of students using a supplement were considered as true positive and vice versa. Participants who were unsure if they used a supplement in the last month were considered as false positive and false negative. The prevalence data were expressed as percentages (%) and in 95% confidence interval ranges. Chi square (χ^2^) test/ Fisher exact test, correlations and regression analyses were applied where applicable. Chi square (χ^2^) test/ Fisher exact test and correlations were utilized to report any associations and relationships respectively, between independent and dependent variables. Regression analyses were used to determine any determinant of DS use.

### Ethics approval and consent

All participants were provided with a written informed consent. The study was approved by Institutional Review Board of Imam Abdulrahman Bin Faisal University, Dammam, Saudi Arabia (#IRB-UGS-2018-05-074).

## Results

A total of 545 female students responded to the study. The Cronbach’s alpha value was 0.634 (N = 6), i.e. acceptable. The mean age of students was 20.56 ± 1.44 years, most students were single (N = 451, 82.8%) and were studying in 3^rd^ year of study (N = 226, 41.5%). More than a third of students (N = 206, 37.8%) were from pharmacy college. Students from health colleges accounted for more than half (N = 361, 66.2%). Most students lived with family (N = 510, 93.6%), had 3–5 siblings (N = 266, 48.8%) and did not have any illness (N = 528, 96.9%). Of those (N = 17) who had some sort of illness, most students (N = 8) had Glucose 6-phosphate dehydrogenase (G_6_PD) deficiency, some (N = 4) had sickle cell disease (SCD), others had diabetes (N = 1), asthma (N = 1), rheumatoid arthritis (N = 1), hypertension (N = 1) and irritable bowel syndrome (N = 1) (Table 1).

**Table 1:** Participants’ information.

Demographic information (N = 545)	N	%
**Marital status**		
Single	451	82.8
Married	92	16.9
Other	2	0.4
**College type**		
Health	361	66.2
Non-health	184	38.8
**College of study**		
Pharmacy	206	37.8
Medicine	26	4.8
Design	27	5
Nursing	30	5.5
Applied Medical Sciences	69	12.5
Engineering	52	9.5
Applied Studies and Community Service	45	8.3
Business	52	9.5
Science	8	1.5
Dentistry	30	5.5
**Year of study**		
Prep year	4	0.7
2^nd^ year	147	27
3^rd^ year	226	41.5
4^th^ year	110	20.2
5^th^ year	58	10.6
**Number of children**		
1–2 children	21	3.9
3 or more children	8	1.5
Married without children	65	11.9
Not applicable	451	82.8
**Number of siblings**		
Between 1–2 siblings	96	17.6
Between 3–5 siblings	266	48.8
Between 6–8 siblings	148	27.2
More than 8 siblings	22	4
No siblings	13	2.4
**Residence**		
Living with family	510	93.6
Living alone (university accommodation)	35	6.4
**Illnesses**		
No, I do not suffer from any illness	528	96.9
Yes	17	3.1

### Prevalence of dietary supplement use

The prevalence of dietary supplement use was 32.70% (95% CI: 29.06%– 36.51%). The prevalence was 29.77% (95% CI: 25.29%– 34.56%) among students at all health colleges combined and similarly, it was 37.50% (95% CI: 31.36%– 43.96%) among students at all non-health colleges. Data for college and year-wise prevalence are presented in Table 2.

**Table 2:** Prevalence of dietary supplement used in colleges and study year.

College of study	Prevalence (%)	95% CI range
Pharmacy	25.78	20.19–32.01
Medicine	30.77	14.13–51.79
Design	23.53	10.75–41.17
Nursing	33.33	17.29–52.81
Applied Medical Sciences	36	25.23–47.91
Engineering	45.78	34.79–57.08
Applied Studies and Community Service	44.44	30.92–58.60
Business	47.44	36.01–59.07
Science	-	-
Dentistry	37.84	22.46–55.24
**Year of study**		
Prep year	-	-
2^nd^ year	23.39	17.27–30.46
3^rd^ year	41.7	35.76–47.82
4^th^ year	17.36	11.08–25.3
5^th^ year	46.03	33.39–59.06

### Types of DS, reasons for use and experience

Of those students who used supplements (N = 181, 33.2%) and assuming it as 100%. A brand was commonly used (N = 31, 17.2%) while some used generic (N = 26, 14.4%) and both brand and generics (N = 16, 8.4%). Most students did not know the type (N = 108, 59.7%). The most common reason for use of supplements was general health and well-being (N = 116, 21.3%) and the most commonly used supplement was multivitamin (N = 167, 30.6%). The majority of students (N = 126, 23.1%) did not suffer from any adverse drug reaction (ADR) that could be attributed to supplement use (Table 3).

**Table 3:** Reasons for use and experience with supplements.

Reasons for DS use	N	%
General Health and Well Being	116	21.3
Boost immunity	9	1.7
Weight gain	3	0.6
Physician’s recommendation	33	6.1
Enhancement of memory	2	0.4
Increase performance/sports	1	0.2
Joint care	2	0.4
Poor diet/malnutrition	7	1.3
Source of energy	3	0.6
More than one reason	25	4.6
Not applicable	344	63.1
**Types of DS used**		
I did not use	348	63.9
Multivitamins	167	30.6
Glucosamine/omega 3 FA	6	1.1
Whey protein	7	1.3
Calcium	5	0.9
More than one supplement	12	2.2
**Experience with DS use**		
Had an ADR attributable to DS use	3	0.6
Had an ADR but not sure if it was attributed to DS use	38	7
No ADR	126	23.1
Not applicable	378	69.4

### Monthly expenditure on supplements

A total of 55 students reported the cost incurred on the use of DS. The average monthly expenditure on procurement of supplements was reported at SAR 286 (USD 76.3), i.e., mean = 286.53 ± 397, while the median cost reported was SAR 100 (USD 26.6), [IQR = 250]. The lowest figure for monthly expenditure was reported at SAR 9 (USD 2.4) while the highest was SAR 1800 (USD 479.9). The value of USD corresponds to SAR to USD exchange rate at the time of this writing.

### Student opinions, attitude, and source of information regarding DS

Students were asked to rate their satisfaction with DS on a scale of 0 (worst) to 5 (best). The average rating was 2.57 ± 1.35 (median = 3). Most students (N = 392, 71.9%) agreed that DS were good for health however, recommended their use based on a physician’s recommendation (N = 474, 87%). Most students (N = 262, 48.1%) had an opinion that using DS regularly could prevent the risk of chronic illness. Most students (N = 297, 54.5%) mentioned healthcare professionals as their source of information regarding DS (Table 4).

**Table 4:** Student opinions, attitude, and source of information regarding DS.

Do you consider DS good for health?	N	%
Agree	392	71.9
I do not know	130	23.9
Disagree	23	4.2
**Encourage the use of DS to others?**		
Always recommend	52	9.5
Based on physician’s recommendation only	474	87
Not at all	19	3.5
**Opinion about DS**		
Necessary for all ages	78	14.3
They are harmless	196	36
Regular use of dietary supplement prevents chronic disease	262	48.1
Dietary supplements may prevent cancer	9	1.7
**Source of information regarding DS**		
Friends, family, and relatives	151	27.7
Healthcare professionals, (physicians, pharmacists, etc.)	297	54.5
Internet	78	14.3
Newspaper and magazine	9	1.7
More than one source	10	1.8

### Association of students’ demographics with their opinion

The demographics of students were cross tabulated with their opinion of DS being good for health. The variable of students’ age was significantly associated (p<0.02) with opinion as most of them who aged 20 or above, agreed that DS are good for health. Further, there was a significant association (p<0.001) between the variables of ‘college’ and ‘opinion’ as students from medicine, design, engineering, applied studies and community service, science and dentistry colleges agreed that DS are good for health while students from pharmacy, nursing, applied medical sciences, business colleges disagreed. As a binary variable, a significant association (p<0.001) existed as students from non-health-cluster colleges agreed with opinion more than their counterparts. Participants who studied in the 3^rd^ year and 5^th^ year had favorable opinion (p<0.001) while those who had a major illness had positive opinion of supplements (p<0.05) (Table 5).

**Table 5:** Cross tabulation between students’ demographic characteristics with opinion.

Characteristics		Opinion (Dietary supplements are good for health)		P value
		Disagree	Agree	
**Age**				0.015
Equal or above 20 years	Count	105	308	
	Expected Count	115.9	297.1	
	% within Age group	25.4%	74.6%	
	% within Opinion	68.6%	78.6%	
Less than 20 years	Count	48	84	
	Expected Count	37.1	94.9	
	% within Age group	36.4%	63.6%	
	% within Opinion	31.4%	21.4%	
**College**				<0.001
Pharmacy	Count	60	146	
	Expected Count	57.8	148.2	
	% within College	29.1%	70.9%	
	% within Opinion	39.2%	37.2%	
Medicine	Count	6	20	
	Expected Count	7.3	18.7	
	% within College	23.1%	76.9%	
	% within Opinion	3.9%	5.1%	
Design	Count	6	21	
	Expected Count	7.6	19.4	
	% within College	22.2%	77.8%	
	% within Opinion	3.9%	5.4%	
Nursing	Count	27	3	
	Expected Count	8.4	21.6	
	% within College	90%	10%	
	% within Opinion	17.6%	.8%	
Applied medical sciences	Count	26	43	
	Expected Count	19.4	49.6	
	% within College	37.7%	62.3%	
	% within Opinion	17%	11%	
Engineering	Count	8	44	
	Expected Count	14.6	37.4	
	% within College	15.4%	84.6%	
	% within Opinion	5.2%	11.2%	
Applied studies and community service	Count	0	45	
	Expected Count	12.6	32.4	
	% within College	0.0%	100%	
	% within Opinion	0.0%	11.5%	
Business	Count	20	32	
	Expected Count	14.6	37.4	
	% within College	38.5%	61.5%	
	% within Opinion	13.1%	8.2%	
Science	Count	0	8	
	Expected Count	2.2	5.8	
	% within College	0%	100%	
	% within Opinion	0%	2%	
Dentistry	Count	0	30	
	Expected Count	8.4	21.6	
	% within College	0%	100%	
	% within Opinion	0%	7.7%	
**College cluster**				<0.001
Health	Count	119	242	
	Expected Count	101.3	259.7	
	% within College cluster	33%	67%	
	% within Opinion	77.8%	61.7%	
Non-health	Count	34	150	
	Expected Count	51.7	132.3	
	% within College cluster	18.5%	81.5%	
	% within Opinion	22.2%	38.3%	
**Study year**				<0.001*
Preparatory year	Count	2	2	
	Expected Count	1.1	2.9	
	% within Study year	50%	50%	
	% within Opinion	1.3%	0.5%	
2nd year	Count	62	85	
	Expected Count	41.3	105.7	
	% within Study year	42.2%	57.8%	
	% within Opinion	40.5%	21.7%	
3rd year	Count	31	195	
	Expected Count	63.4	162.6	
	% within Study year	13.7%	86.3%	
	% within Opinion	20.3%	49.7%	
4th year	Count	43	67	
	Expected Count	30.9	79.1	
	% within Study year	39.1%	60.9%	
	% within Opinion	28.1%	17.1%	
5th year	Count	15	43	
	Expected Count	16.3	41.7	
	% within Study year	25.9%	74.1%	
	% within Opinion	9.8%	11%	
**Marital status**				0.392
Single	Count	130	321	
	Expected Count	126.6	324.4	
	% within Marital status	28.8%	71.2%	
	% within Opinion	85.0%	81.9%	
Married	Count	23	71	
	Expected Count	26.4	67.6	
	% within Marital status	24.5%	75.5%	
	% within Opinion	15%	18.1%	
**Number of children**				0.049
1–2 children	Count	6	15	
	Expected Count	5.9	15.1	
	% within Children	28.6%	71.4%	
	% within Opinion	3.9%	3.8%	
3 or more children	Count	5	3	
	Expected Count	2.2	5.8	
	% within Children	62.5%	37.5%	
	% within Opinion	3.3%	0.8%	
Married without children	Count	12	53	
	Expected Count	18.2	46.8	
	% within Children	18.5%	81.5%	
	% within Opinion	7.8%	13.5%	
Not applicable to me (single)	Count	130	321	
	Expected Count	126.6	324.4	
	% within Children	28.8%	71.2%	
	% within Opinion	85.0%	81.9%	
**Number of Siblings**				0.042
Between 1–2 siblings	Count	25	71	
	Expected Count	27	69	
	% within Siblings	26%	74%	
	% within Opinion	16.3%	18.1%	
Between 3–5 siblings	Count	63	203	
	Expected Count	74.7	191.3	
	% within Siblings	23.7%	76.3%	
	% within Opinion	41.2%	51.8%	
Between 6–8 siblings	Count	56	92	
	Expected Count	41.5	106.5	
	% within Siblings	37.8%	62.2%	
	% within Opinion	36.6%	23.5%	
More than 8 siblings	Count	6	16	
	Expected Count	6.2	15.8	
	% within Siblings	27.3%	72.7%	
	% within Opinion	3.9%	4.1%	
No siblings	Count	3	10	
	Expected Count	3.6	9.4	
	% within Siblings	23.1%	76.9%	
	% within Opinion	2%	2.6%	
**Residence**				0.398
Living with family	Count	141	369	
	Expected Count	143.2	366.8	
	% within Residence	27.6%	72.4%	
	% within Opinion	92.2%	94.1%	
Living alone (University accommodation)	Count	12	23	
	Expected Count	9.8	25.2	
	% within Residence	34.3%	65.7%	
	% within Opinion	7.8%	5.9%	
**Any major illness**				0.039*
Do not suffer from any illness	Count	152	376	
	Expected Count	148.2	379.8	
	% within illness	28.8%	71.2%	
	% within Opinion	99.3%	95.9%	
Suffer from a major illness	Count	1	16	
	Expected Count	4.8	12.2	
	% within illness	5.9%	94.1%	
	% within Opinion	0.7%	4.1%	

*Fisher exact test

Multivariate logistic regression analysis was conducted to identify the determinants of opinion regarding DS use. The adjusted odds ratios were calculated for the dependent variable of ‘opinion that dietary supplements are good for health,’ which had two outcomes, i.e., agree and disagree. The outcome of, ‘disagree’ was selected as a reference category. Based on the analysis, students aged 20 years or more, studying in a non-health college and up to 3^rd^ year of study were roughly 2 times more likely to agree while those who had a major illness were 9 times more likely to agree (Table 6).

**Table 6:** Multiple logistic regression model for determinants of opinion regarding DS use.

Factors	Coefficient	SE	p-value	AOR	95% CI for AOR	
					Lower	Upper
Age						
Less than 20 Years (R)	---	---	---	---	---	---
20 years and more	0.877	0.251	<0.001	2.403	1.468	3.933
College cluster						
Health (R)	---	---	---	---	---	---
Non-health	0.824	.227	<0.001	2.281	1.461	3.561
Year of study						
4^th^ year and above (R)	---	---	---	---	---	---
Up to 3^rd^ Year	0.770	.236	0.001	2.159	1.359	3.431
Number of children						
No children (R)	---	---	---	---	---	---
1–2 children	-0.085	.521	0.870	0.918	0.331	2.550
3 or more children	-1.713	.804	0.033	0.180	0.037	.872
Siblings						
Have siblings (R)	---	---	---	---	---	---
Have no siblings	0.061	.717	0.933	1.062	0.261	4.328
Any major illness						
No (R)	---	---	---	---	---	---
Yes	2.230	1.089	0.041	9.301	1.101	78.563

*R = reference case*, *SE = Standard Error*, *CI = Confidence Interval; AOR = Adjusted odds ratio*.

## Discussion

The prevalence of DS use was 32.7% which was higher than the previously reported figures by Albusalih and colleagues, i.e., 30.5% at this venue [23]. Though, Albusalih et al. reported the figures for multivitamin use only, our results may be higher since other supplements were also considered along with multivitamins. Comparatively, the prevalence reported from this venue was low as a study in university students in Riyadh reported a high prevalence of dietary supplements use, i.e., ≥76.6% [19]. In comparison with literature from regional countries, the prevalence obtained in this study was slightly lower than the prevalence reported among college students from in UAE [24]. It was considerably lower than the figures reported for female pharmacy students in Pakistani universities [2]. However, it was higher than the prevalence reported in Japanese students [25].

We further analyzed the prevalence of college based on health and non-background and observed that the prevalence was higher in students at non-health colleges, i.e., 37.50% compared to their counterparts, i.e., 29.77%. Kobayashi et al. and Moore et al. observed a slightly higher trend of supplements use among students of health track [17, 25]. However, in a local study the difference in DS among female students belonging to health sciences and humanities background was not significant [19]. Though, our prevalence calculation method may have been different, the findings still substantiate the possibility that prevalence may not depend on course subjects. On an individual college level, students from the business college had highest prevalence followed by students from the college of engineering. While evaluating prevalence based on years of study, it was observed that the prevalence increased steadily from 2^nd^ year to 3^rd^ year. The prevalence decreased amongst students in their 4^th^ year of study. The highest prevalence was reported in 5^th^ year of study. In a Japanese study, there was an inclining trend when it came to prevalence based on study years [25].

The most commonly used supplements were multivitamins. This was similar to the findings of a study among female Saudi students as the afore mentioned supplements were commonly used [19, 23]. In another study in female students studying in medicine college at this university, a high prevalence of vitamin and minerals supplement use ≥44% was reported [26]. At the same time, a previous study in male colleges at this university highlighted a high prevalence of multivitamins use [3]. This trend in multivitamin use may have been reflected in this study as well.

Most students reasoned general health and well-being for supplement use. This was in line with the findings of study conducted in male students at this university [3]. Contrastingly, Pakistani undergraduate students reasoned physician recommendation for consuming such products [2]. This reason though, was selected but, by a small number of students in this study. The study by Alfawaz and colleagues in Saudi female students highlighted maintenance of healthy hairs and recovery in an illness/injury, as major reasons for use of such products [19]. Similar reasons were also reported by Nigerian and Indian students [27, 28].

With regards the pharmaceutical dosage form, most students used a brand product. This was similar to the preference indicated by male students in another study [3]. This inclination towards brands may be due to the consumers’ perception towards packaging as studies have reported that consumers prefer pharmaceutical products with attractive packaging that are a characteristic feature of brand products [29]. Besides, Zehra et al. highlighted that in Pakistani pharmaceutical market, brands were considered to have better quality when compared to generics as they have had years of research behind them [30]. Moreover, brand products are reasonably priced unlike generic products; an attribute of pharmaceutical grade supplement that sometimes serve as a proxy indicator for product quality. Further to this, online retail pharmacies in Saudi Arabia usually stock brand products and with an increasing use of procurement of these products via online retail drug marts, the consumers are more likely to use brand supplements products. Most participants reported no adverse reactions related to supplement use. This is of particular importance as it was reported in a study that more than a third of Saudi female students may be unaware of potential adverse effects of supplements [19]. It was observed in a study that students at a medical college in a Nigerian university were aware of dietary supplement use [27]. In this study, we observed that less than 1% of students who used supplements reported that they experience an adverse effect attributed to supplements while the proportion for the same among Japanese students was 7.5% [25].

The current study highlighted an average monthly spending of SAR 286, i.e., (USD 76.3) while Naqvi et al. reported an average cost of SAR 278.9, i.e., (USD 74.4) among males [3]. This highlighted no noteworthy change in spending however, the amount spent was substantial. This could be further clarified if it is compared with students at Pakistani universities who spent USD 13.5 on DS use per month. Most participants considered supplements as good for health. This occurrence was similar to the findings of Radwan et al. [24]. In this study, we observed that students who aged 20 years or more, studied in a non-health college and up to 3^rd^ year of study, were roughly 2 times more likely to agree while those who had a major illness were 9 times more likely to agree that supplements are good for health. However, most students indicated that they recommend supplement use to others solely on physician’s advice. This was similar to the response of students from male students at this university as well as from those in Pakistani academia [2, 3]. Female university students in Riyadh also consumed supplements based on physicians’ prescription [19]. We observed that the most common source of information regarding supplements was healthcare professionals unlike the findings of Alfawaz where social media was reported to be the most common source followed by internet [19]. Moreover, most students in Japan used internet as a source of information for supplements [25].

The study has few limitations such as the use of convenience sampling that substantially limits the generalizability of the results. At the same time, exclusion of the male students renders the study unable to have a gender-based analysis of dietary supplement intake. Further studies are recommended that consider gender perspective in regards to the use of supplements.

## Conclusion

The data highlighted that dietary supplements were commonly used among female students at this university however, it was quite low as compared to students from other local and regional universities. Moreover, the prevalence was higher in non-health colleges as compared to health colleges which was opposite to the previously reported findings among male students at this venue. The most common supplements were brand products and, multivitamins, and used for general health and well-being. A substantial amount of money was spent monthly on supplements. This highlights the inclination of students towards supplement use.

## Supporting information

S1 Dataset (SPV)

S2 Dataset (SPV)

S3 Dataset (SPV)

S4 Dataset (SPV)

S5 Dataset (SPV)

S6 Dataset (SPV)

S1 File (PDF)

## Abbreviations

DS: dietary supplements
DSQ: Dietary Supplement Questionnaire
SPSS: Statistical Package for Social Sciences
USD: United States Dollar
SAR: Saudi Arabian Riyal
UK: United Kingdom
NHANES: National Health and Nutrition Examination Survey
ADE: Adverse drug events
ADR: Adverse drug reaction
IAU: Imam Abdulrahman Bin Faisal University
