# Supplementary material for: Prevalence, reasons, and determinants of dietary supplements use among undergraduate female students of health and non-health colleges in a Saudi public university
Source: PLoS One. 2021 Mar 3;16(3):e0247295. doi: 10.1371/journal.pone.0247295 (PMC7928447; doi:10.1371/journal.pone.0247295)
Supplement: S1 File — (PDF) [file pone.0247295.s007.pdf]

MEDCALC®
easy-to-use statistical software

Result Output Screenshot for Overall Prevalence

Free statistical calculators

Diagnostic test evaluation calculator

Instructions: enter the number of cases in the diseased group that test positive (*a*) and negative (*b*); and the number of cases in the non-diseased group that test positive (*c*) and negative (*d*).

Disease prevalence

If the sample sizes in the positive (Disease present) and the negative (Disease absent) groups do not reflect the real prevalence of the disease, you can enter the disease prevalence (expressed as a percentage) in the corresponding input box.

Next click the **Test** button.

	Disease				
**Test**	**Present**	**n**	**Absent**	**n**	**Total**
**Positive**	True Positive	a= 119	False Positive	c= 88	a + c = **207**
**Negative**	False Negative	b= 88	True Negative	d= 338	b + d = **426**
**Total**		a + b = **207**		c + d = **426**	

Disease prevalence

If the ratio of cases in the Disease Present and Disease Absent groups does not reflect the disease prevalence, enter:

disease prevalence (%):

Test

Results

Statistic	Value	95% CI
Sensitivity	57.49%	50.45% to 64.31%
Specificity	79.34%	75.18% to 83.09%
Positive Likelihood Ratio	2.78	2.23 to 3.47
Negative Likelihood Ratio	0.54	0.45 to 0.63
Disease prevalence (*)	32.70%	29.06% to 36.51%
Positive Predictive Value (*)	57.49%	52.05% to 62.75%
Negative Predictive Value (*)	79.34%	76.50% to 81.93%
Accuracy (*)	72.20%	68.53% to 75.65%

(*) These values are dependent on disease prevalence.

Definitions

- *Sensitivity*: probability that a test result will be positive when the disease is present (true positive rate).

  = a / (a+b)

- *Specificity*: probability that a test result will be negative when the disease is not present (true negative rate).

  = d / (c+d)

- *Positive likelihood ratio*: ratio between the probability of a positive test result given the *presence* of the disease and the probability of a positive test result given the *absence* of the disease, i.e.

  = True positive rate / False positive rate = Sensitivity / (1-Specificity)

- *Negative likelihood ratio*: ratio between the probability of a negative test result given the *presence* of the disease and the probability of a negative test result given the *absence* of the disease, i.e.

  = False negative rate / True negative rate = (1-Sensitivity) / Specificity

- *Positive predictive value*: probability that the disease is present when the test is positive.

$$PPV = \frac{sensitivity \times prevalence}{sensitivity \times prevalence + (1 - specificity) \times (1 - prevalence)}$$

- *Negative predictive value*: probability that the disease is not present when the test is negative.

$$NPV = \frac{specificity \times (1 - prevalence)}{(1 - sensitivity) \times prevalence + specificity \times (1 - prevalence)}$$

- *Accuracy*: overall probability that a patient is correctly classified.

  = Sensitivity × Prevalence + Specificity × (1 − Prevalence)

Sensitivity, specificity, disease prevalence, positive and negative predictive value as well as accuracy are expressed as percentages.

Confidence intervals for sensitivity, specificity and accuracy are "exact" Clopper-Pearson confidence intervals.

Confidence intervals for the likelihood ratios are calculated using the "Log method" as given on page 109 of Altman et al. 2000.

Confidence intervals for the predictive values are the standard logit confidence intervals given by Mercaldo et al. 2007.

Literature

- Altman DG, Machin D, Bryant TN, Gardner MJ (Eds) (2000) Statistics with confidence, 2nd ed. BMJ Books.
- Gardner IA, Greiner M (2006) Receiver-operating characteristic curves and likelihood ratios: improvements over traditional methods for the evaluation and application of veterinary clinical pathology tests. Veterinary Clinical Pathology 35:8-17. PubMed
- Griner PF, Mayewski RJ, Mushlin AI, Greenland P (1981) Selection and interpretation of diagnostic tests and procedures. Annals of Internal Medicine 94:555-600. PubMed
- Hanley JA, McNeil BJ (1982) The meaning and use of the area under a receiver operating characteristic (ROC) curve. Radiology 143:29-36. PubMed
- Mercaldo ND, Lau KF, Zhou XH (2007) Confidence intervals for predictive values with an emphasis to case-control studies. Statistics in Medicine 26:2170-2183. PubMed
- Metz CE (1978) Basic principles of ROC analysis. Seminars in Nuclear Medicine 8:283-298. PubMed
- Zhou XH, NA Obuchowski, DK McClish (2002) Statistical methods in diagnostic medicine. New York: Wiley.
- Zweig MH, Campbell G (1993) Receiver-operating characteristic (ROC) plots: a fundamental evaluation tool in clinical medicine. Clinical Chemistry 39:561-577. PubMed

External links

- Binomial proportion confidence interval on Wikipedia.

Share Tweet

© 2020 MedCalc Software Ltd

MEDCALC®

easy-to-use statistical software

Result Output Screenshot for All health colleges

Free statistical calculators

Diagnostic test evaluation calculator

Instructions: enter the number of cases in the diseased group that test positive (*a*) and negative (*b*); and the number of cases in the non-diseased group that test positive (*c*) and negative (*d*).

Disease prevalence

If the sample sizes in the positive (Disease present) and the negative (Disease absent) groups do not reflect the real prevalence of the disease, you can enter the disease prevalence (expressed as a percentage) in the corresponding input box.

Next click the **Test** button.

Test	Disease: Present	n	Absent	n	Total
**Positive**	True Positive	a= 85	False Positive	c= 32	a + c = **117**
**Negative**	False Negative	b= 32	True Negative	d= 244	b + d = **276**
**Total**		a + b = **117**		c + d = **276**	

Disease prevalence

If the ratio of cases in the Disease Present and Disease Absent groups does not reflect the disease prevalence, enter:

disease prevalence (%):

Test

Results

Statistic	Value	95% CI
Sensitivity	72.65%	63.64% to 80.48%
Specificity	88.41%	84.03% to 91.93%
Positive Likelihood Ratio	6.27	4.44 to 8.84
Negative Likelihood Ratio	0.31	0.23 to 0.42
Disease prevalence (*)	29.77%	25.29% to 34.56%
Positive Predictive Value (*)	72.65%	65.31% to 78.94%
Negative Predictive Value (*)	88.41%	84.98% to 91.13%
Accuracy (*)	83.72%	79.69% to 87.23%

(*) These values are dependent on disease prevalence.

Definitions

- *Sensitivity*: probability that a test result will be positive when the disease is present (true positive rate).

  = a / (a+b)

- *Specificity*: probability that a test result will be negative when the disease is not present (true negative rate).

  = d / (c+d)

- *Positive likelihood ratio*: ratio between the probability of a positive test result given the *presence* of the disease and the probability of a positive test result given the *absence* of the disease, i.e.

  = True positive rate / False positive rate = Sensitivity / (1-Specificity)

- *Negative likelihood ratio*: ratio between the probability of a negative test result given the *presence* of the disease and the probability of a negative test result given the *absence* of the disease, i.e.

  = False negative rate / True negative rate = (1-Sensitivity) / Specificity

- *Positive predictive value*: probability that the disease is present when the test is positive.

$$PPV = \frac{sensitivity \times prevalence}{sensitivity \times prevalence + (1 - specificity) \times (1 - prevalence)}$$

- *Negative predictive value*: probability that the disease is not present when the test is negative.

$$NPV = \frac{specificity \times (1 - prevalence)}{(1 - sensitivity) \times prevalence + specificity \times (1 - prevalence)}$$

- *Accuracy*: overall probability that a patient is correctly classified.

  = Sensitivity × Prevalence + Specificity × (1 − Prevalence)

Sensitivity, specificity, disease prevalence, positive and negative predictive value as well as accuracy are expressed as percentages.

Confidence intervals for sensitivity, specificity and accuracy are "exact" Clopper-Pearson confidence intervals.

Confidence intervals for the likelihood ratios are calculated using the "Log method" as given on page 109 of Altman et al. 2000.

Confidence intervals for the predictive values are the standard logit confidence intervals given by Mercaldo et al. 2007.

Literature

- Altman DG, Machin D, Bryant TN, Gardner MJ (Eds) (2000) Statistics with confidence, 2nd ed. BMJ Books.
- Gardner IA, Greiner M (2006) Receiver-operating characteristic curves and likelihood ratios: improvements over traditional methods for the evaluation and application of veterinary clinical pathology tests. Veterinary Clinical Pathology 35:8-17. PubMed
- Griner PF, Mayewski RJ, Mushlin AI, Greenland P (1981) Selection and interpretation of diagnostic tests and procedures. Annals of Internal Medicine 94:555-600. PubMed
- Hanley JA, McNeil BJ (1982) The meaning and use of the area under a receiver operating characteristic (ROC) curve. Radiology 143:29-36. PubMed
- Mercaldo ND, Lau KF, Zhou XH (2007) Confidence intervals for predictive values with an emphasis to case-control studies. Statistics in Medicine 26:2170-2183. PubMed
- Metz CE (1978) Basic principles of ROC analysis. Seminars in Nuclear Medicine 8:283-298. PubMed
- Zhou XH, NA Obuchowski, DK McClish (2002) Statistical methods in diagnostic medicine. New York: Wiley.
- Zweig MH, Campbell G (1993) Receiver-operating characteristic (ROC) plots: a fundamental evaluation tool in clinical medicine. Clinical Chemistry 39:561-577. PubMed

External links

- Binomial proportion confidence interval on Wikipedia.

Share Tweet

© 2020 MedCalc Software Ltd

MEDCALC®
easy-to-use statistical software

Result Output Screenshot for All non-health colleges

Free statistical calculators

Diagnostic test evaluation calculator

Instructions: enter the number of cases in the diseased group that test positive (*a*) and negative (*b*); and the number of cases in the non-diseased group that test positive (*c*) and negative (*d*).

Disease prevalence

If the sample sizes in the positive (Disease present) and the negative (Disease absent) groups do not reflect the real prevalence of the disease, you can enter the disease prevalence (expressed as a percentage) in the corresponding input box.

Next click the **Test** button.

	Disease				
**Test**	**Present**	**n**	**Absent**	**n**	**Total**
**Positive**	True Positive	a= 34	False Positive	c= 56	a + c = **90**
**Negative**	False Negative	b= 56	True Negative	d= 94	b + d = **150**
**Total**		a + b = **90**		c + d = **150**	

Disease prevalence

If the ratio of cases in the Disease Present and Disease Absent groups does not reflect the disease prevalence, enter:

disease prevalence (%):

Test

Results

**Statistic**	**Value**	**95% CI**
Sensitivity	37.78%	27.77% to 48.62%
Specificity	62.67%	54.40% to 70.42%
Positive Likelihood Ratio	1.01	0.72 to 1.42
Negative Likelihood Ratio	0.99	0.81 to 1.22
Disease prevalence (*)	37.50%	31.36% to 43.96%
Positive Predictive Value (*)	37.78%	30.25% to 45.95%
Negative Predictive Value (*)	62.67%	57.81% to 67.28%
Accuracy (*)	53.33%	46.81% to 59.78%

(*) These values are dependent on disease prevalence.

Definitions

- *Sensitivity*: probability that a test result will be positive when the disease is present (true positive rate).

  = a / (a+b)

- *Specificity*: probability that a test result will be negative when the disease is not present (true negative rate).

  = d / (c+d)

- *Positive likelihood ratio*: ratio between the probability of a positive test result given the *presence* of the disease and the probability of a positive test result given the *absence* of the disease, i.e.

  = True positive rate / False positive rate = Sensitivity / (1-Specificity)

- *Negative likelihood ratio*: ratio between the probability of a negative test result given the *presence* of the disease and the probability of a negative test result given the *absence* of the disease, i.e.

  = False negative rate / True negative rate = (1-Sensitivity) / Specificity

- *Positive predictive value*: probability that the disease is present when the test is positive.

$$PPV = \frac{sensitivity \times prevalence}{sensitivity \times prevalence + (1 - specificity) \times (1 - prevalence)}$$

- *Negative predictive value*: probability that the disease is not present when the test is negative.

$$NPV = \frac{specificity \times (1 - prevalence)}{(1 - sensitivity) \times prevalence + specificity \times (1 - prevalence)}$$

- *Accuracy*: overall probability that a patient is correctly classified.

  = Sensitivity × Prevalence + Specificity × (1 − Prevalence)

Sensitivity, specificity, disease prevalence, positive and negative predictive value as well as accuracy are expressed as percentages.

Confidence intervals for sensitivity, specificity and accuracy are "exact" Clopper-Pearson confidence intervals.

Confidence intervals for the likelihood ratios are calculated using the "Log method" as given on page 109 of Altman et al. 2000.

Confidence intervals for the predictive values are the standard logit confidence intervals given by Mercaldo et al. 2007.

Literature

- Altman DG, Machin D, Bryant TN, Gardner MJ (Eds) (2000) Statistics with confidence, 2nd ed. BMJ Books.
- Gardner IA, Greiner M (2006) Receiver-operating characteristic curves and likelihood ratios: improvements over traditional methods for the evaluation and application of veterinary clinical pathology tests. Veterinary Clinical Pathology 35:8-17. PubMed
- Griner PF, Mayewski RJ, Mushlin AI, Greenland P (1981) Selection and interpretation of diagnostic tests and procedures. Annals of Internal Medicine 94:555-600. PubMed
- Hanley JA, McNeil BJ (1982) The meaning and use of the area under a receiver operating characteristic (ROC) curve. Radiology 143:29-36. PubMed
- Mercaldo ND, Lau KF, Zhou XH (2007) Confidence intervals for predictive values with an emphasis to case-control studies. Statistics in Medicine 26:2170-2183. PubMed
- Metz CE (1978) Basic principles of ROC analysis. Seminars in Nuclear Medicine 8:283-298. PubMed
- Zhou XH, NA Obuchowski, DK McClish (2002) Statistical methods in diagnostic medicine. New York: Wiley.
- Zweig MH, Campbell G (1993) Receiver-operating characteristic (ROC) plots: a fundamental evaluation tool in clinical medicine. Clinical Chemistry 39:561-577. PubMed

External links

- Binomial proportion confidence interval on Wikipedia.

Share Tweet

© 2020 MedCalc Software Ltd

MEDCALC®
easy-to-use statistical software

Result Output Screenshot for Pharmacy College

Free statistical calculators

Diagnostic test evaluation calculator

Instructions: enter the number of cases in the diseased group that test positive (*a*) and negative (*b*); and the number of cases in the non-diseased group that test positive (*c*) and negative (*d*).

Disease prevalence

If the sample sizes in the positive (Disease present) and the negative (Disease absent) groups do not reflect the real prevalence of the disease, you can enter the disease prevalence (expressed as a percentage) in the corresponding input box.

Next click the **Test** button.

	**Disease**				
**Test**	**Present**	**n**	**Absent**	**n**	**Total**
**Positive**	True Positive	a= 39	False Positive	c= 19	a + c = **58**
**Negative**	False Negative	b= 19	True Negative	d= 148	b + d = **167**
**Total**		a + b = **58**		c + d = **167**	

Disease prevalence

If the ratio of cases in the Disease Present and Disease Absent groups does not reflect the disease prevalence, enter:

disease prevalence (%):

Test

Results

**Statistic**	**Value**	**95% CI**
Sensitivity	67.24%	53.66% to 78.99%
Specificity	88.62%	82.80% to 93.01%
Positive Likelihood Ratio	5.91	3.73 to 9.36
Negative Likelihood Ratio	0.37	0.25 to 0.54
Disease prevalence (*)	25.78%	20.19% to 32.01%
Positive Predictive Value (*)	67.24%	56.45% to 76.48%
Negative Predictive Value (*)	88.62%	84.29% to 91.87%
Accuracy (*)	83.11%	77.56% to 87.76%

(*) These values are dependent on disease prevalence.

Definitions

- *Sensitivity*: probability that a test result will be positive when the disease is present (true positive rate).

  = a / (a+b)

- *Specificity*: probability that a test result will be negative when the disease is not present (true negative rate).

  = d / (c+d)

- *Positive likelihood ratio*: ratio between the probability of a positive test result given the *presence* of the disease and the probability of a positive test result given the *absence* of the disease, i.e.

  = True positive rate / False positive rate = Sensitivity / (1-Specificity)

- *Negative likelihood ratio*: ratio between the probability of a negative test result given the *presence* of the disease and the probability of a negative test result given the *absence* of the disease, i.e.

  = False negative rate / True negative rate = (1-Sensitivity) / Specificity

- *Positive predictive value*: probability that the disease is present when the test is positive.

$$PPV = \frac{sensitivity \times prevalence}{sensitivity \times prevalence + (1 - specificity) \times (1 - prevalence)}$$

- *Negative predictive value*: probability that the disease is not present when the test is negative.

$$NPV = \frac{specificity \times (1 - prevalence)}{(1 - sensitivity) \times prevalence + specificity \times (1 - prevalence)}$$

- *Accuracy*: overall probability that a patient is correctly classified.

  = Sensitivity × Prevalence + Specificity × (1 − Prevalence)

Sensitivity, specificity, disease prevalence, positive and negative predictive value as well as accuracy are expressed as percentages.

Confidence intervals for sensitivity, specificity and accuracy are "exact" Clopper-Pearson confidence intervals.

Confidence intervals for the likelihood ratios are calculated using the "Log method" as given on page 109 of Altman et al. 2000.

Confidence intervals for the predictive values are the standard logit confidence intervals given by Mercaldo et al. 2007.

Literature

- Altman DG, Machin D, Bryant TN, Gardner MJ (Eds) (2000) Statistics with confidence, 2nd ed. BMJ Books.
- Gardner IA, Greiner M (2006) Receiver-operating characteristic curves and likelihood ratios: improvements over traditional methods for the evaluation and application of veterinary clinical pathology tests. Veterinary Clinical Pathology 35:8-17. PubMed
- Griner PF, Mayewski RJ, Mushlin AI, Greenland P (1981) Selection and interpretation of diagnostic tests and procedures. Annals of Internal Medicine 94:555-600. PubMed
- Hanley JA, McNeil BJ (1982) The meaning and use of the area under a receiver operating characteristic (ROC) curve. Radiology 143:29-36. PubMed
- Mercaldo ND, Lau KF, Zhou XH (2007) Confidence intervals for predictive values with an emphasis to case-control studies. Statistics in Medicine 26:2170-2183. PubMed
- Metz CE (1978) Basic principles of ROC analysis. Seminars in Nuclear Medicine 8:283-298. PubMed
- Zhou XH, NA Obuchowski, DK McClish (2002) Statistical methods in diagnostic medicine. New York: Wiley.
- Zweig MH, Campbell G (1993) Receiver-operating characteristic (ROC) plots: a fundamental evaluation tool in clinical medicine. Clinical Chemistry 39:561-577. PubMed

External links

- Binomial proportion confidence interval on Wikipedia.

Share Tweet

© 2020 MedCalc Software Ltd

MEDCALC®
easy-to-use statistical software

Result Output Screenshot for College of Medicine

Free statistical calculators

Diagnostic test evaluation calculator

Instructions: enter the number of cases in the diseased group that test positive (*a*) and negative (*b*); and the number of cases in the non-diseased group that test positive (*c*) and negative (*d*).

Disease prevalence

If the sample sizes in the positive (Disease present) and the negative (Disease absent) groups do not reflect the real prevalence of the disease, you can enter the disease prevalence (expressed as a percentage) in the corresponding input box.

Next click the **Test** button.

	Disease				
**Test**	**Present**	**n**	**Absent**	**n**	**Total**
**Positive**	True Positive	a= 8	False Positive	c= 0	a + c = **8**
**Negative**	False Negative	b= 0	True Negative	d= 18	b + d = **18**
**Total**		a + b = **8**		c + d = **18**	

Disease prevalence

If the ratio of cases in the Disease Present and Disease Absent groups does not reflect the disease prevalence, enter:

disease prevalence (%):

Test

Results

Statistic	Value	95% CI
Sensitivity	100.00%	63.06% to 100.00%
Specificity	100.00%	81.47% to 100.00%
Positive Likelihood Ratio		
Negative Likelihood Ratio	0.00	
Disease prevalence (*)	30.77%	14.33% to 51.79%
Positive Predictive Value (*)	100.00%	
Negative Predictive Value (*)	100.00%	
Accuracy (*)	100.00%	86.77% to 100.00%

(*) These values are dependent on disease prevalence.

Definitions

- *Sensitivity*: probability that a test result will be positive when the disease is present (true positive rate).

  = a / (a+b)

- *Specificity*: probability that a test result will be negative when the disease is not present (true negative rate).

  = d / (c+d)

- *Positive likelihood ratio*: ratio between the probability of a positive test result given the *presence* of the disease and the probability of a positive test result given the *absence* of the disease, i.e.

  = True positive rate / False positive rate = Sensitivity / (1-Specificity)

- *Negative likelihood ratio*: ratio between the probability of a negative test result given the *presence* of the disease and the probability of a negative test result given the *absence* of the disease, i.e.

  = False negative rate / True negative rate = (1-Sensitivity) / Specificity

- *Positive predictive value*: probability that the disease is present when the test is positive.

$$PPV = \frac{sensitivity \times prevalence}{sensitivity \times prevalence + (1 - specificity) \times (1 - prevalence)}$$

- *Negative predictive value*: probability that the disease is not present when the test is negative.

$$NPV = \frac{specificity \times (1 - prevalence)}{(1 - sensitivity) \times prevalence + specificity \times (1 - prevalence)}$$

- *Accuracy*: overall probability that a patient is correctly classified.

  = Sensitivity × Prevalence + Specificity × (1 − Prevalence)

Sensitivity, specificity, disease prevalence, positive and negative predictive value as well as accuracy are expressed as percentages.

Confidence intervals for sensitivity, specificity and accuracy are "exact" Clopper-Pearson confidence intervals.

Confidence intervals for the likelihood ratios are calculated using the "Log method" as given on page 109 of Altman et al. 2000.

Confidence intervals for the predictive values are the standard logit confidence intervals given by Mercaldo et al. 2007.

Literature

- Altman DG, Machin D, Bryant TN, Gardner MJ (Eds) (2000) Statistics with confidence, 2nd ed. BMJ Books.
- Gardner IA, Greiner M (2006) Receiver-operating characteristic curves and likelihood ratios: improvements over traditional methods for the evaluation and application of veterinary clinical pathology tests. Veterinary Clinical Pathology 35:8-17. PubMed
- Griner PF, Mayewski RJ, Mushlin AI, Greenland P (1981) Selection and interpretation of diagnostic tests and procedures. Annals of Internal Medicine 94:555-600. PubMed
- Hanley JA, McNeil BJ (1982) The meaning and use of the area under a receiver operating characteristic (ROC) curve. Radiology 143:29-36. PubMed
- Mercaldo ND, Lau KF, Zhou XH (2007) Confidence intervals for predictive values with an emphasis to case-control studies. Statistics in Medicine 26:2170-2183. PubMed
- Metz CE (1978) Basic principles of ROC analysis. Seminars in Nuclear Medicine 8:283-298. PubMed
- Zhou XH, NA Obuchowski, DK McClish (2002) Statistical methods in diagnostic medicine. New York: Wiley.
- Zweig MH, Campbell G (1993) Receiver-operating characteristic (ROC) plots: a fundamental evaluation tool in clinical medicine. Clinical Chemistry 39:561-577. PubMed

External links

- Binomial proportion confidence interval on Wikipedia.

Share Tweet

© 2020 MedCalc Software Ltd

MEDCALC®
easy-to-use statistical software

Result Output Screenshot for College of Design

Free statistical calculators

Diagnostic test evaluation calculator

Instructions: enter the number of cases in the diseased group that test positive (*a*) and negative (*b*); and the number of cases in the non-diseased group that test positive (*c*) and negative (*d*).

Disease prevalence

If the sample sizes in the positive (Disease present) and the negative (Disease absent) groups do not reflect the real prevalence of the disease, you can enter the disease prevalence (expressed as a percentage) in the corresponding input box.

Next click the **Test** button.

	Disease				
**Test**	**Present**	**n**	**Absent**	**n**	**Total**
**Positive**	True Positive	a= 1	False Positive	c= 7	a + c = **8**
**Negative**	False Negative	b= 7	True Negative	d= 19	b + d = **26**
**Total**		a + b = **8**		c + d = **26**	

Disease prevalence

If the ratio of cases in the Disease Present and Disease Absent groups does not reflect the disease prevalence, enter:

disease prevalence (%):

Test

Results

**Statistic**	**Value**	**95% CI**
Sensitivity	12.50%	0.32% to 52.65%
Specificity	73.08%	52.21% to 88.43%
Positive Likelihood Ratio	0.46	0.07 to 3.23
Negative Likelihood Ratio	1.20	0.84 to 1.70
Disease prevalence (*)	23.53%	10.75% to 41.17%
Positive Predictive Value (*)	12.50%	2.01% to 49.84%
Negative Predictive Value (*)	73.08%	65.65% to 79.40%
Accuracy (*)	58.82%	40.70% to 75.35%

(*) These values are dependent on disease prevalence.

Definitions

- *Sensitivity*: probability that a test result will be positive when the disease is present (true positive rate).

  = a / (a+b)

- *Specificity*: probability that a test result will be negative when the disease is not present (true negative rate).

  = d / (c+d)

- *Positive likelihood ratio*: ratio between the probability of a positive test result given the *presence* of the disease and the probability of a positive test result given the *absence* of the disease, i.e.

  = True positive rate / False positive rate = Sensitivity / (1-Specificity)

- *Negative likelihood ratio*: ratio between the probability of a negative test result given the *presence* of the disease and the probability of a negative test result given the *absence* of the disease, i.e.

  = False negative rate / True negative rate = (1-Sensitivity) / Specificity

- *Positive predictive value*: probability that the disease is present when the test is positive.

$$PPV = \frac{sensitivity \times prevalence}{sensitivity \times prevalence + (1 - specificity) \times (1 - prevalence)}$$

- *Negative predictive value*: probability that the disease is not present when the test is negative.

$$NPV = \frac{specificity \times (1 - prevalence)}{(1 - sensitivity) \times prevalence + specificity \times (1 - prevalence)}$$

- *Accuracy*: overall probability that a patient is correctly classified.

  = Sensitivity × Prevalence + Specificity × (1 − Prevalence)

Sensitivity, specificity, disease prevalence, positive and negative predictive value as well as accuracy are expressed as percentages.

Confidence intervals for sensitivity, specificity and accuracy are "exact" Clopper-Pearson confidence intervals.

Confidence intervals for the likelihood ratios are calculated using the "Log method" as given on page 109 of Altman et al. 2000.

Confidence intervals for the predictive values are the standard logit confidence intervals given by Mercaldo et al. 2007.

Literature

- Altman DG, Machin D, Bryant TN, Gardner MJ (Eds) (2000) Statistics with confidence, 2nd ed. BMJ Books.
- Gardner IA, Greiner M (2006) Receiver-operating characteristic curves and likelihood ratios: improvements over traditional methods for the evaluation and application of veterinary clinical pathology tests. Veterinary Clinical Pathology 35:8-17. PubMed
- Griner PF, Mayewski RJ, Mushlin AI, Greenland P (1981) Selection and interpretation of diagnostic tests and procedures. Annals of Internal Medicine 94:555-600. PubMed
- Hanley JA, McNeil BJ (1982) The meaning and use of the area under a receiver operating characteristic (ROC) curve. Radiology 143:29-36. PubMed
- Mercaldo ND, Lau KF, Zhou XH (2007) Confidence intervals for predictive values with an emphasis to case-control studies. Statistics in Medicine 26:2170-2183. PubMed
- Metz CE (1978) Basic principles of ROC analysis. Seminars in Nuclear Medicine 8:283-298. PubMed
- Zhou XH, NA Obuchowski, DK McClish (2002) Statistical methods in diagnostic medicine. New York: Wiley.
- Zweig MH, Campbell G (1993) Receiver-operating characteristic (ROC) plots: a fundamental evaluation tool in clinical medicine. Clinical Chemistry 39:561-577. PubMed

External links

- Binomial proportion confidence interval on Wikipedia.

Share Tweet

© 2020 MedCalc Software Ltd

MEDCALC®
easy-to-use statistical software

Result Output Screenshot for College of Nursing

Free statistical calculators

Diagnostic test evaluation calculator

Instructions: enter the number of cases in the diseased group that test positive (*a*) and negative (*b*); and the number of cases in the non-diseased group that test positive (*c*) and negative (*d*).

Disease prevalence

If the sample sizes in the positive (Disease present) and the negative (Disease absent) groups do not reflect the real prevalence of the disease, you can enter the disease prevalence (expressed as a percentage) in the corresponding input box.

Next click the **Test** button.

	Disease				
**Test**	**Present**	**n**	**Absent**	**n**	**Total**
**Positive**	True Positive	a= 10	False Positive	c= 0	a + c = **10**
**Negative**	False Negative	b= 0	True Negative	d= 20	b + d = **20**
**Total**		a + b = **10**		c + d = **20**	

Disease prevalence

If the ratio of cases in the Disease Present and Disease Absent groups does not reflect the disease prevalence, enter:

disease prevalence (%):

Test

Results

Statistic	Value	95% CI
Sensitivity	100.00%	69.15% to 100.00%
Specificity	100.00%	83.16% to 100.00%
Positive Likelihood Ratio		
Negative Likelihood Ratio	0.00	
Disease prevalence (*)	33.33%	17.29% to 52.81%
Positive Predictive Value (*)	100.00%	
Negative Predictive Value (*)	100.00%	
Accuracy (*)	100.00%	88.43% to 100.00%

(*) These values are dependent on disease prevalence.

Definitions

- *Sensitivity*: probability that a test result will be positive when the disease is present (true positive rate).

  = a / (a+b)

- *Specificity*: probability that a test result will be negative when the disease is not present (true negative rate).

  = d / (c+d)

- *Positive likelihood ratio*: ratio between the probability of a positive test result given the *presence* of the disease and the probability of a positive test result given the *absence* of the disease, i.e.

  = True positive rate / False positive rate = Sensitivity / (1-Specificity)

- *Negative likelihood ratio*: ratio between the probability of a negative test result given the *presence* of the disease and the probability of a negative test result given the *absence* of the disease, i.e.

  = False negative rate / True negative rate = (1-Sensitivity) / Specificity

- *Positive predictive value*: probability that the disease is present when the test is positive.

$$PPV = \frac{sensitivity \times prevalence}{sensitivity \times prevalence + (1 - specificity) \times (1 - prevalence)}$$

- *Negative predictive value*: probability that the disease is not present when the test is negative.

$$NPV = \frac{specificity \times (1 - prevalence)}{(1 - sensitivity) \times prevalence + specificity \times (1 - prevalence)}$$

- *Accuracy*: overall probability that a patient is correctly classified.

  = Sensitivity × Prevalence + Specificity × (1 − Prevalence)

Sensitivity, specificity, disease prevalence, positive and negative predictive value as well as accuracy are expressed as percentages.

Confidence intervals for sensitivity, specificity and accuracy are "exact" Clopper-Pearson confidence intervals.

Confidence intervals for the likelihood ratios are calculated using the "Log method" as given on page 109 of Altman et al. 2000.

Confidence intervals for the predictive values are the standard logit confidence intervals given by Mercaldo et al. 2007.

Literature

- Altman DG, Machin D, Bryant TN, Gardner MJ (Eds) (2000) Statistics with confidence, 2nd ed. BMJ Books.
- Gardner IA, Greiner M (2006) Receiver-operating characteristic curves and likelihood ratios: improvements over traditional methods for the evaluation and application of veterinary clinical pathology tests. Veterinary Clinical Pathology 35:8-17. PubMed
- Griner PF, Mayewski RJ, Mushlin AI, Greenland P (1981) Selection and interpretation of diagnostic tests and procedures. Annals of Internal Medicine 94:555-600. PubMed
- Hanley JA, McNeil BJ (1982) The meaning and use of the area under a receiver operating characteristic (ROC) curve. Radiology 143:29-36. PubMed
- Mercaldo ND, Lau KF, Zhou XH (2007) Confidence intervals for predictive values with an emphasis to case-control studies. Statistics in Medicine 26:2170-2183. PubMed
- Metz CE (1978) Basic principles of ROC analysis. Seminars in Nuclear Medicine 8:283-298. PubMed
- Zhou XH, NA Obuchowski, DK McClish (2002) Statistical methods in diagnostic medicine. New York: Wiley.
- Zweig MH, Campbell G (1993) Receiver-operating characteristic (ROC) plots: a fundamental evaluation tool in clinical medicine. Clinical Chemistry 39:561-577. PubMed

External links

- Binomial proportion confidence interval on Wikipedia.

Share Tweet

© 2020 MedCalc Software Ltd

MEDCALC®
easy-to-use statistical software

Result Output Screenshot for College of Applied Medical Science

Free statistical calculators

Diagnostic test evaluation calculator

Instructions: enter the number of cases in the diseased group that test positive (*a*) and negative (*b*); and the number of cases in the non-diseased group that test positive (*c*) and negative (*d*).

Disease prevalence

If the sample sizes in the positive (Disease present) and the negative (Disease absent) groups do not reflect the real prevalence of the disease, you can enter the disease prevalence (expressed as a percentage) in the corresponding input box.

Next click the **Test** button.

	**Disease**				
**Test**	**Present**	**n**	**Absent**	**n**	**Total**
**Positive**	True Positive	a= 21	False Positive	c= 6	a + c = **27**
**Negative**	False Negative	b= 6	True Negative	d= 42	b + d = **48**
**Total**		a + b = **27**		c + d = **48**	

Disease prevalence

If the ratio of cases in the Disease Present and Disease Absent groups does not reflect the disease prevalence, enter:

disease prevalence (%):

Test

Results

**Statistic**	**Value**	**95% CI**
Sensitivity	77.78%	57.74% to 91.38%
Specificity	87.50%	74.75% to 95.27%
Positive Likelihood Ratio	6.22	2.87 to 13.51
Negative Likelihood Ratio	0.25	0.12 to 0.52
Disease prevalence (*)	36.00%	25.23% to 47.91%
Positive Predictive Value (*)	77.78%	61.72% to 88.37%
Negative Predictive Value (*)	87.50%	77.42% to 93.46%
Accuracy (*)	84.00%	73.72% to 91.45%

(*) These values are dependent on disease prevalence.

Definitions

- *Sensitivity*: probability that a test result will be positive when the disease is present (true positive rate).

  = a / (a+b)

- *Specificity*: probability that a test result will be negative when the disease is not present (true negative rate).

  = d / (c+d)

- *Positive likelihood ratio*: ratio between the probability of a positive test result given the *presence* of the disease and the probability of a positive test result given the *absence* of the disease, i.e.

  = True positive rate / False positive rate = Sensitivity / (1-Specificity)

- *Negative likelihood ratio*: ratio between the probability of a negative test result given the *presence* of the disease and the probability of a negative test result given the *absence* of the disease, i.e.

  = False negative rate / True negative rate = (1-Sensitivity) / Specificity

- *Positive predictive value*: probability that the disease is present when the test is positive.

$$PPV = \frac{sensitivity \times prevalence}{sensitivity \times prevalence + (1 - specificity) \times (1 - prevalence)}$$

- *Negative predictive value*: probability that the disease is not present when the test is negative.

$$NPV = \frac{specificity \times (1 - prevalence)}{(1 - sensitivity) \times prevalence + specificity \times (1 - prevalence)}$$

- *Accuracy*: overall probability that a patient is correctly classified.

  = Sensitivity × Prevalence + Specificity × (1 − Prevalence)

Sensitivity, specificity, disease prevalence, positive and negative predictive value as well as accuracy are expressed as percentages.

Confidence intervals for sensitivity, specificity and accuracy are "exact" Clopper-Pearson confidence intervals.

Confidence intervals for the likelihood ratios are calculated using the "Log method" as given on page 109 of Altman et al. 2000.

Confidence intervals for the predictive values are the standard logit confidence intervals given by Mercaldo et al. 2007.

Literature

- Altman DG, Machin D, Bryant TN, Gardner MJ (Eds) (2000) Statistics with confidence, 2nd ed. BMJ Books.
- Gardner IA, Greiner M (2006) Receiver-operating characteristic curves and likelihood ratios: improvements over traditional methods for the evaluation and application of veterinary clinical pathology tests. Veterinary Clinical Pathology 35:8-17. PubMed
- Griner PF, Mayewski RJ, Mushlin AI, Greenland P (1981) Selection and interpretation of diagnostic tests and procedures. Annals of Internal Medicine 94:555-600. PubMed
- Hanley JA, McNeil BJ (1982) The meaning and use of the area under a receiver operating characteristic (ROC) curve. Radiology 143:29-36. PubMed
- Mercaldo ND, Lau KF, Zhou XH (2007) Confidence intervals for predictive values with an emphasis to case-control studies. Statistics in Medicine 26:2170-2183. PubMed
- Metz CE (1978) Basic principles of ROC analysis. Seminars in Nuclear Medicine 8:283-298. PubMed
- Zhou XH, NA Obuchowski, DK McClish (2002) Statistical methods in diagnostic medicine. New York: Wiley.
- Zweig MH, Campbell G (1993) Receiver-operating characteristic (ROC) plots: a fundamental evaluation tool in clinical medicine. Clinical Chemistry 39:561-577. PubMed

External links

- Binomial proportion confidence interval on Wikipedia.

Share Tweet

© 2020 MedCalc Software Ltd

MEDCALC®
easy-to-use statistical software

Result Output Screenshot for College of Engineering

Free statistical calculators

Diagnostic test evaluation calculator

Instructions: enter the number of cases in the diseased group that test positive (*a*) and negative (*b*); and the number of cases in the non-diseased group that test positive (*c*) and negative (*d*).

Disease prevalence

If the sample sizes in the positive (Disease present) and the negative (Disease absent) groups do not reflect the real prevalence of the disease, you can enter the disease prevalence (expressed as a percentage) in the corresponding input box.

Next click the **Test** button.

	Disease				
**Test**	**Present**	**n**	**Absent**	**n**	**Total**
**Positive**	True Positive	a= 7	False Positive	c= 31	a + c = **38**
**Negative**	False Negative	b= 31	True Negative	d= 14	b + d = **45**
**Total**		a + b = **38**		c + d = **45**	

Disease prevalence

If the ratio of cases in the Disease Present and Disease Absent groups does not reflect the disease prevalence, enter:

disease prevalence (%):

Test

Results

Statistic	Value	95% CI
Sensitivity	18.42%	7.74% to 34.33%
Specificity	31.11%	18.17% to 46.65%
Positive Likelihood Ratio	0.27	0.13 to 0.54
Negative Likelihood Ratio	2.62	1.65 to 4.15
Disease prevalence (*)	45.78%	34.79% to 57.08%
Positive Predictive Value (*)	18.42%	10.11% to 31.20%
Negative Predictive Value (*)	31.11%	22.18% to 41.71%
Accuracy (*)	25.30%	16.39% to 36.04%

(*) These values are dependent on disease prevalence.

Definitions

- *Sensitivity*: probability that a test result will be positive when the disease is present (true positive rate).

  = a / (a+b)

- *Specificity*: probability that a test result will be negative when the disease is not present (true negative rate).

  = d / (c+d)

- *Positive likelihood ratio*: ratio between the probability of a positive test result given the *presence* of the disease and the probability of a positive test result given the *absence* of the disease, i.e.

  = True positive rate / False positive rate = Sensitivity / (1-Specificity)

- *Negative likelihood ratio*: ratio between the probability of a negative test result given the *presence* of the disease and the probability of a negative test result given the *absence* of the disease, i.e.

  = False negative rate / True negative rate = (1-Sensitivity) / Specificity

- *Positive predictive value*: probability that the disease is present when the test is positive.

$$PPV = \frac{sensitivity \times prevalence}{sensitivity \times prevalence + (1 - specificity) \times (1 - prevalence)}$$

- *Negative predictive value*: probability that the disease is not present when the test is negative.

$$NPV = \frac{specificity \times (1 - prevalence)}{(1 - sensitivity) \times prevalence + specificity \times (1 - prevalence)}$$

- *Accuracy*: overall probability that a patient is correctly classified.

  = Sensitivity × Prevalence + Specificity × (1 − Prevalence)

Sensitivity, specificity, disease prevalence, positive and negative predictive value as well as accuracy are expressed as percentages.

Confidence intervals for sensitivity, specificity and accuracy are "exact" Clopper-Pearson confidence intervals.

Confidence intervals for the likelihood ratios are calculated using the "Log method" as given on page 109 of Altman et al. 2000.

Confidence intervals for the predictive values are the standard logit confidence intervals given by Mercaldo et al. 2007.

Literature

- Altman DG, Machin D, Bryant TN, Gardner MJ (Eds) (2000) Statistics with confidence, 2nd ed. BMJ Books.
- Gardner IA, Greiner M (2006) Receiver-operating characteristic curves and likelihood ratios: improvements over traditional methods for the evaluation and application of veterinary clinical pathology tests. Veterinary Clinical Pathology 35:8-17. PubMed
- Griner PF, Mayewski RJ, Mushlin AI, Greenland P (1981) Selection and interpretation of diagnostic tests and procedures. Annals of Internal Medicine 94:555-600. PubMed
- Hanley JA, McNeil BJ (1982) The meaning and use of the area under a receiver operating characteristic (ROC) curve. Radiology 143:29-36. PubMed
- Mercaldo ND, Lau KF, Zhou XH (2007) Confidence intervals for predictive values with an emphasis to case-control studies. Statistics in Medicine 26:2170-2183. PubMed
- Metz CE (1978) Basic principles of ROC analysis. Seminars in Nuclear Medicine 8:283-298. PubMed
- Zhou XH, NA Obuchowski, DK McClish (2002) Statistical methods in diagnostic medicine. New York: Wiley.
- Zweig MH, Campbell G (1993) Receiver-operating characteristic (ROC) plots: a fundamental evaluation tool in clinical medicine. Clinical Chemistry 39:561-577. PubMed

External links

- Binomial proportion confidence interval on Wikipedia.

Share Tweet

© 2020 MedCalc Software Ltd

MEDCALC®
easy-to-use statistical software

Result Output Screenshot for
College of Applied Studies and Community Service

Free statistical calculators

Diagnostic test evaluation calculator

Instructions: enter the number of cases in the diseased group that test positive (*a*) and negative (*b*); and the number of cases in the non-diseased group that test positive (*c*) and negative (*d*).

Disease prevalence

If the sample sizes in the positive (Disease present) and the negative (Disease absent) groups do not reflect the real prevalence of the disease, you can enter the disease prevalence (expressed as a percentage) in the corresponding input box.

Next click the **Test** button.

	**Disease**				
**Test**	**Present**	**n**	**Absent**	**n**	**Total**
**Positive**	True Positive	a= 15	False Positive	c= 9	a + c = **24**
**Negative**	False Negative	b= 9	True Negative	d= 21	b + d = **30**
**Total**		a + b = **24**		c + d = **30**	

Disease prevalence

If the ratio of cases in the Disease Present and Disease Absent groups does not reflect the disease prevalence, enter:

disease prevalence (%):

Test

Results

Statistic	Value	95% CI
Sensitivity	62.50%	40.59% to 81.20%
Specificity	70.00%	50.60% to 85.27%
Positive Likelihood Ratio	2.08	1.11 to 3.91
Negative Likelihood Ratio	0.54	0.30 to 0.94
Disease prevalence (*)	44.44%	30.92% to 58.60%
Positive Predictive Value (*)	62.50%	47.07% to 75.75%
Negative Predictive Value (*)	70.00%	56.96% to 80.45%
Accuracy (*)	66.67%	52.53% to 78.91%

(*) These values are dependent on disease prevalence.

Definitions

- *Sensitivity*: probability that a test result will be positive when the disease is present (true positive rate).

  = a / (a+b)

- *Specificity*: probability that a test result will be negative when the disease is not present (true negative rate).

  = d / (c+d)

- *Positive likelihood ratio*: ratio between the probability of a positive test result given the *presence* of the disease and the probability of a positive test result given the *absence* of the disease, i.e.

  = True positive rate / False positive rate = Sensitivity / (1-Specificity)

- *Negative likelihood ratio*: ratio between the probability of a negative test result given the *presence* of the disease and the probability of a negative test result given the *absence* of the disease, i.e.

  = False negative rate / True negative rate = (1-Sensitivity) / Specificity

- *Positive predictive value*: probability that the disease is present when the test is positive.

$$PPV = \frac{sensitivity \times prevalence}{sensitivity \times prevalence + (1 - specificity) \times (1 - prevalence)}$$

- *Negative predictive value*: probability that the disease is not present when the test is negative.

$$NPV = \frac{specificity \times (1 - prevalence)}{(1 - sensitivity) \times prevalence + specificity \times (1 - prevalence)}$$

- *Accuracy*: overall probability that a patient is correctly classified.

  = Sensitivity × Prevalence + Specificity × (1 − Prevalence)

Sensitivity, specificity, disease prevalence, positive and negative predictive value as well as accuracy are expressed as percentages.

Confidence intervals for sensitivity, specificity and accuracy are "exact" Clopper-Pearson confidence intervals.

Confidence intervals for the likelihood ratios are calculated using the "Log method" as given on page 109 of Altman et al. 2000.

Confidence intervals for the predictive values are the standard logit confidence intervals given by Mercaldo et al. 2007.

Literature

- Altman DG, Machin D, Bryant TN, Gardner MJ (Eds) (2000) Statistics with confidence, 2nd ed. BMJ Books.
- Gardner IA, Greiner M (2006) Receiver-operating characteristic curves and likelihood ratios: improvements over traditional methods for the evaluation and application of veterinary clinical pathology tests. Veterinary Clinical Pathology 35:8-17. PubMed
- Griner PF, Mayewski RJ, Mushlin AI, Greenland P (1981) Selection and interpretation of diagnostic tests and procedures. Annals of Internal Medicine 94:555-600. PubMed
- Hanley JA, McNeil BJ (1982) The meaning and use of the area under a receiver operating characteristic (ROC) curve. Radiology 143:29-36. PubMed
- Mercaldo ND, Lau KF, Zhou XH (2007) Confidence intervals for predictive values with an emphasis to case-control studies. Statistics in Medicine 26:2170-2183. PubMed
- Metz CE (1978) Basic principles of ROC analysis. Seminars in Nuclear Medicine 8:283-298. PubMed
- Zhou XH, NA Obuchowski, DK McClish (2002) Statistical methods in diagnostic medicine. New York: Wiley.
- Zweig MH, Campbell G (1993) Receiver-operating characteristic (ROC) plots: a fundamental evaluation tool in clinical medicine. Clinical Chemistry 39:561-577. PubMed

External links

- Binomial proportion confidence interval on Wikipedia.

Share Tweet

© 2020 MedCalc Software Ltd

MEDCALC®
easy-to-use statistical software

Result Output Screenshot for College of Business

Free statistical calculators

Diagnostic test evaluation calculator

Instructions: enter the number of cases in the diseased group that test positive (*a*) and negative (*b*); and the number of cases in the non-diseased group that test positive (*c*) and negative (*d*).

Disease prevalence

If the sample sizes in the positive (Disease present) and the negative (Disease absent) groups do not reflect the real prevalence of the disease, you can enter the disease prevalence (expressed as a percentage) in the corresponding input box.

Next click the **Test** button.

	Disease				
**Test**	**Present**	**n**	**Absent**	**n**	**Total**
**Positive**	True Positive	a= 11	False Positive	c= 26	a + c = **37**
**Negative**	False Negative	b= 26	True Negative	d= 15	b + d = **41**
**Total**		a + b = **37**		c + d = **41**	

Disease prevalence

If the ratio of cases in the Disease Present and Disease Absent groups does not reflect the disease prevalence, enter:

disease prevalence (%):

Test

Results

Statistic	Value	95% CI
Sensitivity	29.73%	15.87% to 46.98%
Specificity	36.59%	22.12% to 53.06%
Positive Likelihood Ratio	0.47	0.27 to 0.81
Negative Likelihood Ratio	1.92	1.22 to 3.03
Disease prevalence (*)	47.44%	36.01% to 59.07%
Positive Predictive Value (*)	29.73%	19.66% to 42.24%
Negative Predictive Value (*)	36.59%	26.81% to 47.61%
Accuracy (*)	33.33%	23.06% to 44.92%

(*) These values are dependent on disease prevalence.

Definitions

- *Sensitivity*: probability that a test result will be positive when the disease is present (true positive rate).

  = a / (a+b)

- *Specificity*: probability that a test result will be negative when the disease is not present (true negative rate).

  = d / (c+d)

- *Positive likelihood ratio*: ratio between the probability of a positive test result given the *presence* of the disease and the probability of a positive test result given the *absence* of the disease, i.e.

  = True positive rate / False positive rate = Sensitivity / (1-Specificity)

- *Negative likelihood ratio*: ratio between the probability of a negative test result given the *presence* of the disease and the probability of a negative test result given the *absence* of the disease, i.e.

  = False negative rate / True negative rate = (1-Sensitivity) / Specificity

- *Positive predictive value*: probability that the disease is present when the test is positive.

$$PPV = \frac{sensitivity \times prevalence}{sensitivity \times prevalence + (1 - specificity) \times (1 - prevalence)}$$

- *Negative predictive value*: probability that the disease is not present when the test is negative.

$$NPV = \frac{specificity \times (1 - prevalence)}{(1 - sensitivity) \times prevalence + specificity \times (1 - prevalence)}$$

- *Accuracy*: overall probability that a patient is correctly classified.

  = Sensitivity × Prevalence + Specificity × (1 − Prevalence)

Sensitivity, specificity, disease prevalence, positive and negative predictive value as well as accuracy are expressed as percentages.

Confidence intervals for sensitivity, specificity and accuracy are "exact" Clopper-Pearson confidence intervals.

Confidence intervals for the likelihood ratios are calculated using the "Log method" as given on page 109 of Altman et al. 2000.

Confidence intervals for the predictive values are the standard logit confidence intervals given by Mercaldo et al. 2007.

Literature

- Altman DG, Machin D, Bryant TN, Gardner MJ (Eds) (2000) Statistics with confidence, 2nd ed. BMJ Books.
- Gardner IA, Greiner M (2006) Receiver-operating characteristic curves and likelihood ratios: improvements over traditional methods for the evaluation and application of veterinary clinical pathology tests. Veterinary Clinical Pathology 35:8-17. PubMed
- Griner PF, Mayewski RJ, Mushlin AI, Greenland P (1981) Selection and interpretation of diagnostic tests and procedures. Annals of Internal Medicine 94:555-600. PubMed
- Hanley JA, McNeil BJ (1982) The meaning and use of the area under a receiver operating characteristic (ROC) curve. Radiology 143:29-36. PubMed
- Mercaldo ND, Lau KF, Zhou XH (2007) Confidence intervals for predictive values with an emphasis to case-control studies. Statistics in Medicine 26:2170-2183. PubMed
- Metz CE (1978) Basic principles of ROC analysis. Seminars in Nuclear Medicine 8:283-298. PubMed
- Zhou XH, NA Obuchowski, DK McClish (2002) Statistical methods in diagnostic medicine. New York: Wiley.
- Zweig MH, Campbell G (1993) Receiver-operating characteristic (ROC) plots: a fundamental evaluation tool in clinical medicine. Clinical Chemistry 39:561-577. PubMed

External links

- Binomial proportion confidence interval on Wikipedia.

Share Tweet

© 2020 MedCalc Software Ltd

MEDCALC®
easy-to-use statistical software

Result Output Screenshot for College of Dentistry

Free statistical calculators

Diagnostic test evaluation calculator

Instructions: enter the number of cases in the diseased group that test positive (*a*) and negative (*b*); and the number of cases in the non-diseased group that test positive (*c*) and negative (*d*).

Disease prevalence

If the sample sizes in the positive (Disease present) and the negative (Disease absent) groups do not reflect the real prevalence of the disease, you can enter the disease prevalence (expressed as a percentage) in the corresponding input box.

Next click the **Test** button.

	**Disease**				
**Test**	**Present**	**n**	**Absent**	**n**	**Total**
**Positive**	True Positive	a= 7	False Positive	c= 7	a + c = **14**
**Negative**	False Negative	b= 7	True Negative	d= 16	b + d = **23**
**Total**		a + b = **14**		c + d = **23**	

Disease prevalence

If the ratio of cases in the Disease Present and Disease Absent groups does not reflect the disease prevalence, enter:

disease prevalence (%):

Test

Results

Statistic	Value	95% CI
Sensitivity	50.00%	23.04% to 76.96%
Specificity	69.57%	47.08% to 86.79%
Positive Likelihood Ratio	1.64	0.73 to 3.69
Negative Likelihood Ratio	0.72	0.40 to 1.30
Disease prevalence (*)	37.84%	22.46% to 55.24%
Positive Predictive Value (*)	50.00%	30.79% to 69.21%
Negative Predictive Value (*)	69.57%	55.90% to 80.47%
Accuracy (*)	62.16%	44.76% to 77.54%

(*) These values are dependent on disease prevalence.

Definitions

- *Sensitivity*: probability that a test result will be positive when the disease is present (true positive rate).

  = a / (a+b)

- *Specificity*: probability that a test result will be negative when the disease is not present (true negative rate).

  = d / (c+d)

- *Positive likelihood ratio*: ratio between the probability of a positive test result given the *presence* of the disease and the probability of a positive test result given the *absence* of the disease, i.e.

  = True positive rate / False positive rate = Sensitivity / (1-Specificity)

- *Negative likelihood ratio*: ratio between the probability of a negative test result given the *presence* of the disease and the probability of a negative test result given the *absence* of the disease, i.e.

  = False negative rate / True negative rate = (1-Sensitivity) / Specificity

- *Positive predictive value*: probability that the disease is present when the test is positive.

$$PPV = \frac{sensitivity \times prevalence}{sensitivity \times prevalence + (1 - specificity) \times (1 - prevalence)}$$

- *Negative predictive value*: probability that the disease is not present when the test is negative.

$$NPV = \frac{specificity \times (1 - prevalence)}{(1 - sensitivity) \times prevalence + specificity \times (1 - prevalence)}$$

- *Accuracy*: overall probability that a patient is correctly classified.

  = Sensitivity × Prevalence + Specificity × (1 − Prevalence)

Sensitivity, specificity, disease prevalence, positive and negative predictive value as well as accuracy are expressed as percentages.

Confidence intervals for sensitivity, specificity and accuracy are "exact" Clopper-Pearson confidence intervals.

Confidence intervals for the likelihood ratios are calculated using the "Log method" as given on page 109 of Altman et al. 2000.

Confidence intervals for the predictive values are the standard logit confidence intervals given by Mercaldo et al. 2007.

Literature

- Altman DG, Machin D, Bryant TN, Gardner MJ (Eds) (2000) Statistics with confidence, 2nd ed. BMJ Books.
- Gardner IA, Greiner M (2006) Receiver-operating characteristic curves and likelihood ratios: improvements over traditional methods for the evaluation and application of veterinary clinical pathology tests. Veterinary Clinical Pathology 35:8-17. PubMed
- Griner PF, Mayewski RJ, Mushlin AI, Greenland P (1981) Selection and interpretation of diagnostic tests and procedures. Annals of Internal Medicine 94:555-600. PubMed
- Hanley JA, McNeil BJ (1982) The meaning and use of the area under a receiver operating characteristic (ROC) curve. Radiology 143:29-36. PubMed
- Mercaldo ND, Lau KF, Zhou XH (2007) Confidence intervals for predictive values with an emphasis to case-control studies. Statistics in Medicine 26:2170-2183. PubMed
- Metz CE (1978) Basic principles of ROC analysis. Seminars in Nuclear Medicine 8:283-298. PubMed
- Zhou XH, NA Obuchowski, DK McClish (2002) Statistical methods in diagnostic medicine. New York: Wiley.
- Zweig MH, Campbell G (1993) Receiver-operating characteristic (ROC) plots: a fundamental evaluation tool in clinical medicine. Clinical Chemistry 39:561-577. PubMed

External links

- Binomial proportion confidence interval on Wikipedia.

Share Tweet

© 2020 MedCalc Software Ltd

MEDCALC®
easy-to-use statistical software

Result Output Screenshot for 2nd year students

Free statistical calculators

Diagnostic test evaluation calculator

Instructions: enter the number of cases in the diseased group that test positive (*a*) and negative (*b*); and the number of cases in the non-diseased group that test positive (*c*) and negative (*d*).

Disease prevalence

If the sample sizes in the positive (Disease present) and the negative (Disease absent) groups do not reflect the real prevalence of the disease, you can enter the disease prevalence (expressed as a percentage) in the corresponding input box.

Next click the **Test** button.

	**Disease**				
**Test**	**Present**	**n**	**Absent**	**n**	**Total**
**Positive**	True Positive	a= 16	False Positive	c= 24	a + c = **40**
**Negative**	False Negative	b= 24	True Negative	d= 107	b + d = **131**
**Total**		a + b = **40**		c + d = **131**	

Disease prevalence

If the ratio of cases in the Disease Present and Disease Absent groups does not reflect the disease prevalence, enter:

disease prevalence (%):

Test

Results

Statistic	Value	95% CI
Sensitivity	40.00%	24.86% to 56.67%
Specificity	81.68%	73.98% to 87.89%
Positive Likelihood Ratio	2.18	1.29 to 3.69
Negative Likelihood Ratio	0.73	0.56 to 0.96
Disease prevalence (*)	23.39%	17.27% to 30.46%
Positive Predictive Value (*)	40.00%	28.30% to 52.97%
Negative Predictive Value (*)	81.68%	77.37% to 85.33%
Accuracy (*)	71.93%	64.56% to 78.52%

(*) These values are dependent on disease prevalence.

Definitions

- *Sensitivity*: probability that a test result will be positive when the disease is present (true positive rate).

  = a / (a+b)

- *Specificity*: probability that a test result will be negative when the disease is not present (true negative rate).

  = d / (c+d)

- *Positive likelihood ratio*: ratio between the probability of a positive test result given the *presence* of the disease and the probability of a positive test result given the *absence* of the disease, i.e.

  = True positive rate / False positive rate = Sensitivity / (1-Specificity)

- *Negative likelihood ratio*: ratio between the probability of a negative test result given the *presence* of the disease and the probability of a negative test result given the *absence* of the disease, i.e.

  = False negative rate / True negative rate = (1-Sensitivity) / Specificity

- *Positive predictive value*: probability that the disease is present when the test is positive.

$$PPV = \frac{sensitivity \times prevalence}{sensitivity \times prevalence + (1 - specificity) \times (1 - prevalence)}$$

- *Negative predictive value*: probability that the disease is not present when the test is negative.

$$NPV = \frac{specificity \times (1 - prevalence)}{(1 - sensitivity) \times prevalence + specificity \times (1 - prevalence)}$$

- *Accuracy*: overall probability that a patient is correctly classified.

  = Sensitivity × Prevalence + Specificity × (1 − Prevalence)

Sensitivity, specificity, disease prevalence, positive and negative predictive value as well as accuracy are expressed as percentages.

Confidence intervals for sensitivity, specificity and accuracy are "exact" Clopper-Pearson confidence intervals.

Confidence intervals for the likelihood ratios are calculated using the "Log method" as given on page 109 of Altman et al. 2000.

Confidence intervals for the predictive values are the standard logit confidence intervals given by Mercaldo et al. 2007.

Literature

- Altman DG, Machin D, Bryant TN, Gardner MJ (Eds) (2000) Statistics with confidence, 2nd ed. BMJ Books.
- Gardner IA, Greiner M (2006) Receiver-operating characteristic curves and likelihood ratios: improvements over traditional methods for the evaluation and application of veterinary clinical pathology tests. Veterinary Clinical Pathology 35:8-17. PubMed
- Griner PF, Mayewski RJ, Mushlin AI, Greenland P (1981) Selection and interpretation of diagnostic tests and procedures. Annals of Internal Medicine 94:555-600. PubMed
- Hanley JA, McNeil BJ (1982) The meaning and use of the area under a receiver operating characteristic (ROC) curve. Radiology 143:29-36. PubMed
- Mercaldo ND, Lau KF, Zhou XH (2007) Confidence intervals for predictive values with an emphasis to case-control studies. Statistics in Medicine 26:2170-2183. PubMed
- Metz CE (1978) Basic principles of ROC analysis. Seminars in Nuclear Medicine 8:283-298. PubMed
- Zhou XH, NA Obuchowski, DK McClish (2002) Statistical methods in diagnostic medicine. New York: Wiley.
- Zweig MH, Campbell G (1993) Receiver-operating characteristic (ROC) plots: a fundamental evaluation tool in clinical medicine. Clinical Chemistry 39:561-577. PubMed

External links

- Binomial proportion confidence interval on Wikipedia.

Share Tweet

© 2020 MedCalc Software Ltd

MEDCALC®
easy-to-use statistical software

Result Output Screenshot for 3rd Year students

Free statistical calculators

Diagnostic test evaluation calculator

Instructions: enter the number of cases in the diseased group that test positive (*a*) and negative (*b*); and the number of cases in the non-diseased group that test positive (*c*) and negative (*d*).

Disease prevalence

If the sample sizes in the positive (Disease present) and the negative (Disease absent) groups do not reflect the real prevalence of the disease, you can enter the disease prevalence (expressed as a percentage) in the corresponding input box.

Next click the **Test** button.

	Disease				
**Test**	**Present**	**n**	**Absent**	**n**	**Total**
**Positive**	True Positive	a= 68	False Positive	c= 45	a + c = **113**
**Negative**	False Negative	b= 45	True Negative	d= 113	b + d = **158**
**Total**		a + b = **113**		c + d = **158**	

Disease prevalence

If the ratio of cases in the Disease Present and Disease Absent groups does not reflect the disease prevalence, enter:

disease prevalence (%):

Test

Results

Statistic	Value	95% CI
Sensitivity	60.18%	50.54% to 69.27%
Specificity	71.52%	63.80% to 78.41%
Positive Likelihood Ratio	2.11	1.58 to 2.82
Negative Likelihood Ratio	0.56	0.43 to 0.71
Disease prevalence (*)	41.70%	35.76% to 47.82%
Positive Predictive Value (*)	60.18%	53.09% to 66.86%
Negative Predictive Value (*)	71.52%	66.23% to 76.28%
Accuracy (*)	66.79%	60.84% to 72.37%

(*) These values are dependent on disease prevalence.

Definitions

- *Sensitivity*: probability that a test result will be positive when the disease is present (true positive rate).

  = a / (a+b)

- *Specificity*: probability that a test result will be negative when the disease is not present (true negative rate).

  = d / (c+d)

- *Positive likelihood ratio*: ratio between the probability of a positive test result given the *presence* of the disease and the probability of a positive test result given the *absence* of the disease, i.e.

  = True positive rate / False positive rate = Sensitivity / (1-Specificity)

- *Negative likelihood ratio*: ratio between the probability of a negative test result given the *presence* of the disease and the probability of a negative test result given the *absence* of the disease, i.e.

  = False negative rate / True negative rate = (1-Sensitivity) / Specificity

- *Positive predictive value*: probability that the disease is present when the test is positive.

$$PPV = \frac{sensitivity \times prevalence}{sensitivity \times prevalence + (1 - specificity) \times (1 - prevalence)}$$

- *Negative predictive value*: probability that the disease is not present when the test is negative.

$$NPV = \frac{specificity \times (1 - prevalence)}{(1 - sensitivity) \times prevalence + specificity \times (1 - prevalence)}$$

- *Accuracy*: overall probability that a patient is correctly classified.

  = Sensitivity × Prevalence + Specificity × (1 − Prevalence)

Sensitivity, specificity, disease prevalence, positive and negative predictive value as well as accuracy are expressed as percentages.

Confidence intervals for sensitivity, specificity and accuracy are "exact" Clopper-Pearson confidence intervals.

Confidence intervals for the likelihood ratios are calculated using the "Log method" as given on page 109 of Altman et al. 2000.

Confidence intervals for the predictive values are the standard logit confidence intervals given by Mercaldo et al. 2007.

Literature

- Altman DG, Machin D, Bryant TN, Gardner MJ (Eds) (2000) Statistics with confidence, 2nd ed. BMJ Books.
- Gardner IA, Greiner M (2006) Receiver-operating characteristic curves and likelihood ratios: improvements over traditional methods for the evaluation and application of veterinary clinical pathology tests. Veterinary Clinical Pathology 35:8-17.
- Griner PF, Mayewski RJ, Mushlin AI, Greenland P (1981) Selection and interpretation of diagnostic tests and procedures. Annals of Internal Medicine 94:555-600.
- Hanley JA, McNeil BJ (1982) The meaning and use of the area under a receiver operating characteristic (ROC) curve. Radiology 143:29-36.
- Mercaldo ND, Lau KF, Zhou XH (2007) Confidence intervals for predictive values with an emphasis to case-control studies. Statistics in Medicine 26:2170-2183.
- Metz CE (1978) Basic principles of ROC analysis. Seminars in Nuclear Medicine 8:283-298.
- Zhou XH, NA Obuchowski, DK McClish (2002) Statistical methods in diagnostic medicine. New York: Wiley.
- Zweig MH, Campbell G (1993) Receiver-operating characteristic (ROC) plots: a fundamental evaluation tool in clinical medicine. Clinical Chemistry 39:561-577.

External links

- Binomial proportion confidence interval on Wikipedia.

Share Tweet

© 2020 MedCalc Software Ltd

MEDCALC®
easy-to-use statistical software

Result Output Screenshot for 4th year students

Free statistical calculators

Diagnostic test evaluation calculator

Instructions: enter the number of cases in the diseased group that test positive (*a*) and negative (*b*); and the number of cases in the non-diseased group that test positive (*c*) and negative (*d*).

Disease prevalence

If the sample sizes in the positive (Disease present) and the negative (Disease absent) groups do not reflect the real prevalence of the disease, you can enter the disease prevalence (expressed as a percentage) in the corresponding input box.

Next click the **Test** button.

	Disease				
**Test**	**Present**	**n**	**Absent**	**n**	**Total**
**Positive**	True Positive	a= 10	False Positive	c= 11	a + c = **21**
**Negative**	False Negative	b= 11	True Negative	d= 89	b + d = **100**
**Total**		a + b = **21**		c + d = **100**	

Disease prevalence

If the ratio of cases in the Disease Present and Disease Absent groups does not reflect the disease prevalence, enter:

disease prevalence (%):

Test

Results

Statistic	Value	95% CI
Sensitivity	47.62%	25.71% to 70.22%
Specificity	89.00%	81.17% to 94.38%
Positive Likelihood Ratio	4.33	2.12 to 8.85
Negative Likelihood Ratio	0.59	0.39 to 0.89
Disease prevalence (*)	17.36%	11.08% to 25.30%
Positive Predictive Value (*)	47.62%	30.77% to 65.03%
Negative Predictive Value (*)	89.00%	84.25% to 92.44%
Accuracy (*)	81.82%	73.78% to 88.24%

(*) These values are dependent on disease prevalence.

Definitions

- *Sensitivity*: probability that a test result will be positive when the disease is present (true positive rate).

  = a / (a+b)

- *Specificity*: probability that a test result will be negative when the disease is not present (true negative rate).

  = d / (c+d)

- *Positive likelihood ratio*: ratio between the probability of a positive test result given the *presence* of the disease and the probability of a positive test result given the *absence* of the disease, i.e.

  = True positive rate / False positive rate = Sensitivity / (1-Specificity)

- *Negative likelihood ratio*: ratio between the probability of a negative test result given the *presence* of the disease and the probability of a negative test result given the *absence* of the disease, i.e.

  = False negative rate / True negative rate = (1-Sensitivity) / Specificity

- *Positive predictive value*: probability that the disease is present when the test is positive.

$$PPV = \frac{sensitivity \times prevalence}{sensitivity \times prevalence + (1 - specificity) \times (1 - prevalence)}$$

- *Negative predictive value*: probability that the disease is not present when the test is negative.

$$NPV = \frac{specificity \times (1 - prevalence)}{(1 - sensitivity) \times prevalence + specificity \times (1 - prevalence)}$$

- *Accuracy*: overall probability that a patient is correctly classified.

  = Sensitivity × Prevalence + Specificity × (1 − Prevalence)

Sensitivity, specificity, disease prevalence, positive and negative predictive value as well as accuracy are expressed as percentages.

Confidence intervals for sensitivity, specificity and accuracy are "exact" Clopper-Pearson confidence intervals.

Confidence intervals for the likelihood ratios are calculated using the "Log method" as given on page 109 of Altman et al. 2000.

Confidence intervals for the predictive values are the standard logit confidence intervals given by Mercaldo et al. 2007.

Literature

- Altman DG, Machin D, Bryant TN, Gardner MJ (Eds) (2000) Statistics with confidence, 2nd ed. BMJ Books.
- Gardner IA, Greiner M (2006) Receiver-operating characteristic curves and likelihood ratios: improvements over traditional methods for the evaluation and application of veterinary clinical pathology tests. Veterinary Clinical Pathology 35:8-17. PubMed
- Griner PF, Mayewski RJ, Mushlin AI, Greenland P (1981) Selection and interpretation of diagnostic tests and procedures. Annals of Internal Medicine 94:555-600. PubMed
- Hanley JA, McNeil BJ (1982) The meaning and use of the area under a receiver operating characteristic (ROC) curve. Radiology 143:29-36. PubMed
- Mercaldo ND, Lau KF, Zhou XH (2007) Confidence intervals for predictive values with an emphasis to case-control studies. Statistics in Medicine 26:2170-2183. PubMed
- Metz CE (1978) Basic principles of ROC analysis. Seminars in Nuclear Medicine 8:283-298. PubMed
- Zhou XH, NA Obuchowski, DK McClish (2002) Statistical methods in diagnostic medicine. New York: Wiley.
- Zweig MH, Campbell G (1993) Receiver-operating characteristic (ROC) plots: a fundamental evaluation tool in clinical medicine. Clinical Chemistry 39:561-577. PubMed

External links

- Binomial proportion confidence interval on Wikipedia.

Share Tweet

© 2020 MedCalc Software Ltd

MEDCALC®
easy-to-use statistical software

Result Output Screenshot for 5th year students

Free statistical calculators

Diagnostic test evaluation calculator

Instructions: enter the number of cases in the diseased group that test positive (*a*) and negative (*b*); and the number of cases in the non-diseased group that test positive (*c*) and negative (*d*).

Disease prevalence

If the sample sizes in the positive (Disease present) and the negative (Disease absent) groups do not reflect the real prevalence of the disease, you can enter the disease prevalence (expressed as a percentage) in the corresponding input box.

Next click the **Test** button.

	Disease				
**Test**	**Present**	**n**	**Absent**	**n**	**Total**
**Positive**	True Positive	a= 24	False Positive	c= 5	a + c = **29**
**Negative**	False Negative	b= 5	True Negative	d= 29	b + d = **34**
**Total**		a + b = **29**		c + d = **34**	

Disease prevalence

If the ratio of cases in the Disease Present and Disease Absent groups does not reflect the disease prevalence, enter:

disease prevalence (%):

Test

Results

Statistic	Value	95% CI
Sensitivity	82.76%	64.23% to 94.15%
Specificity	85.29%	68.94% to 95.05%
Positive Likelihood Ratio	5.63	2.46 to 12.86
Negative Likelihood Ratio	0.20	0.09 to 0.45
Disease prevalence (*)	46.03%	33.39% to 59.06%
Positive Predictive Value (*)	82.76%	67.75% to 91.64%
Negative Predictive Value (*)	85.29%	72.08% to 92.87%
Accuracy (*)	84.13%	72.74% to 92.12%

(*) These values are dependent on disease prevalence.

Definitions

- *Sensitivity*: probability that a test result will be positive when the disease is present (true positive rate).

  = a / (a+b)

- *Specificity*: probability that a test result will be negative when the disease is not present (true negative rate).

  = d / (c+d)

- *Positive likelihood ratio*: ratio between the probability of a positive test result given the *presence* of the disease and the probability of a positive test result given the *absence* of the disease, i.e.

  = True positive rate / False positive rate = Sensitivity / (1-Specificity)

- *Negative likelihood ratio*: ratio between the probability of a negative test result given the *presence* of the disease and the probability of a negative test result given the *absence* of the disease, i.e.

  = False negative rate / True negative rate = (1-Sensitivity) / Specificity

- *Positive predictive value*: probability that the disease is present when the test is positive.

$$PPV = \frac{sensitivity \times prevalence}{sensitivity \times prevalence + (1 - specificity) \times (1 - prevalence)}$$

- *Negative predictive value*: probability that the disease is not present when the test is negative.

$$NPV = \frac{specificity \times (1 - prevalence)}{(1 - sensitivity) \times prevalence + specificity \times (1 - prevalence)}$$

- *Accuracy*: overall probability that a patient is correctly classified.

  = Sensitivity × Prevalence + Specificity × (1 − Prevalence)

Sensitivity, specificity, disease prevalence, positive and negative predictive value as well as accuracy are expressed as percentages.

Confidence intervals for sensitivity, specificity and accuracy are "exact" Clopper-Pearson confidence intervals.

Confidence intervals for the likelihood ratios are calculated using the "Log method" as given on page 109 of Altman et al. 2000.

Confidence intervals for the predictive values are the standard logit confidence intervals given by Mercaldo et al. 2007.

Literature

- Altman DG, Machin D, Bryant TN, Gardner MJ (Eds) (2000) Statistics with confidence, 2nd ed. BMJ Books.
- Gardner IA, Greiner M (2006) Receiver-operating characteristic curves and likelihood ratios: improvements over traditional methods for the evaluation and application of veterinary clinical pathology tests. Veterinary Clinical Pathology 35:8-17. PubMed
- Griner PF, Mayewski RJ, Mushlin AI, Greenland P (1981) Selection and interpretation of diagnostic tests and procedures. Annals of Internal Medicine 94:555-600. PubMed
- Hanley JA, McNeil BJ (1982) The meaning and use of the area under a receiver operating characteristic (ROC) curve. Radiology 143:29-36. PubMed
- Mercaldo ND, Lau KF, Zhou XH (2007) Confidence intervals for predictive values with an emphasis to case-control studies. Statistics in Medicine 26:2170-2183. PubMed
- Metz CE (1978) Basic principles of ROC analysis. Seminars in Nuclear Medicine 8:283-298. PubMed
- Zhou XH, NA Obuchowski, DK McClish (2002) Statistical methods in diagnostic medicine. New York: Wiley.
- Zweig MH, Campbell G (1993) Receiver-operating characteristic (ROC) plots: a fundamental evaluation tool in clinical medicine. Clinical Chemistry 39:561-577. PubMed

External links

- Binomial proportion confidence interval on Wikipedia.

Share Tweet

© 2020 MedCalc Software Ltd
